# Reciprocal associations between peer problems and non‐suicidal self‐injury throughout adolescence

**DOI:** 10.1111/jcpp.13601

**Published:** 2022-04-05

**Authors:** Lisa De Luca, Matteo Giletta, Ersilia Menesini, Mitchell J. Prinstein

**Affiliations:** ^1^ 9300 Department of Education, Languages Intercultures, Literatures and Psychology University of Florence Firenze Italy; ^2^ 26656 Department of Developmental, Personality and Social Psychology Ghent University Gent Belgium; ^3^ Department of Developmental Psychology Tilburg University Tilburg The Netherlands; ^4^ Department of Psychology and Neuroscience University of North Carolina at Chapel Hill Chapel Hill NC USA

**Keywords:** Adolescence, self‐injury, friendship, bullying peer relationships

## Abstract

**Background:**

Peer problems have emerged as important predictors of Non‐Suicidal Self‐Injury (NSSI) development during adolescence. However, the possibility that adolescents who engage in NSSI may, in turn, be at increased risk for experiencing difficulties with their peers has rarely been examined. This study investigated the reciprocal associations between peer problems (e.g. peer victimization, friendship stress and loneliness) and NSSI throughout adolescence, distinguishing between‐ and within‐person effects.

**Method:**

Participants were 866 adolescents (54.5% females; *M*_age_ = 13.12 years, *SD* = 0.78), who took part in six waves of data collection. Adolescents completed self‐report measures of NSSI, friendship stress and loneliness and they took part in a peer nomination procedure to assess peer victimization. Random Intercept Cross‐Lagged Panel Models (RI‐CLPMs) were used to estimate within‐person cross‐lagged effects between each peer problem and NSSI from Grade 7 to 12.

**Results:**

After accounting for between‐person associations between peer problems and NSSI, results indicated that higher‐than‐usual levels of NSSI predicted higher‐than‐usual levels of adolescents’ own friendship stress, loneliness and peer victimization at the subsequent time point. Yet, sensitivity analyses revealed that most of these effects were strongly attenuated and explained by within‐person fluctuations in depressive symptoms. No within‐person cross‐lagged effects from peer problems to NSSI were found.

**Conclusions:**

Findings highlight that the associations between peer problems (i.e. friendship stress, loneliness) and NSSI may be largely explained by shared underlying factors; yet, some evidence also suggests that NSSI engagement may increase adolescents’ risk to experience difficulties in the relationships with their peers, in part via increases in depressive symptoms.

## Introduction

Non‐suicidal self‐injury (NSSI), defined as the direct and deliberate self‐inflicted damage of body tissue without suicidal intent, is a serious public health concern worldwide, especially during adolescence. The onset of NSSI typically occurs between the ages of 11 and 15 years (Rodav, Levy, & Hamdan, 2014), and approximately 23% of adolescents report to deliberately injuring themselves at least once in their life, 19% in the previous year (Gillies et al., 2018). Prior work has emphasized that during adolescence difficulties with peers (i.e. peer problems) are important precipitants of NSSI development (Prinstein, Guerry, Browne, & Rancourt, 2009). Yet, the possibility that adolescents who engage in NSSI may, in turn, be at increased risk for experiencing problems with their peers has rarely received empirical attention. This is surprising given extensive theoretical work from developmental psychopathology posing possible transactional effects between social environmental factors and individual mental health problems (Rudolph, Lansford, & Rodkin, 2016). Understanding whether peer problems and NSSI mutually reinforce one another over time has significant practical relevance, to prevent that these effects intensify and result in a negative vicious cycle difficult to break. This study, therefore, aimed to investigate the reciprocal associations between peer problems (e.g. peer victimization, friendship stress and loneliness) and NSSI, using a six‐wave prospective design in a large community sample of adolescents.

### Peer problems as antecedents of NSSI

With the transition to adolescence, youth become highly oriented towards their peers, and they show increased sensitivity to both positive as well as negative peer cues (Nelson, Jarcho, & Guyer, 2016). Thus, for adolescents who experience problems with their peers, for example, those exposed to peer victimization, low friendship support or other peer‐related stressors, heightened peer sensitivity may negatively impact socio‐emotional development. Accordingly, a vast peer relations literature has demonstrated that peer problems are associated, concurrently as well as longitudinally, with difficulties in other domains of functioning as well as symptoms of psychopathology (e.g. depression, aggression; Prinstein & Giletta, 2016). Interpersonal models of NSSI also suggest that peer problems may be important risk factors for the development and maintenance of NSSI (Prinstein et al., 2009). According to these models, for individuals who experience stressful and adverse life events, such as peer problems, NSSI may represent a maladaptive coping strategy, for example, to down‐regulate arising negative feelings or to communicate with others (e.g. Liu, Cheek, & Nestor, 2016).

In this study, we focus on three distinct forms of peer problems: peer victimization, friendship stress and loneliness. Peer victimization, referring to being the target of aggressive acts by peers, is among the most stressful experiences youth may be exposed to and, not surprisingly, has been linked to NSSI (Van Geel, Goemans, & Vedder, 2015). In fact, existing longitudinal studies suggest that adolescents exposed to peer victimization are at increased risk for NSSI engagement over time (Cheek, Reiter‐Lavery, & Goldston, 2020). Yet, not only extreme forms of peer problems such as victimization, but also difficulties within positive dyadic relationships or lacking supportive and intimate friendships may be source of interpersonal stress, and could therefore precipitate NSSI (e.g. Giletta et al., 2015). For instance, adolescents who experience conflicts with friends or who report high negative interactions within close relationships (e.g. with romantic partners, friends) have been shown to report higher NSSI (e.g. Hankin & Abela, 2011; Nock & Prinstein, 2004). Therefore, high levels of friendship stress may also increase the risk for NSSI.

Finally, adolescents who struggle to form and maintain positive relationships with their peers, or who are unsatisfied with their peer relationships, tend to report higher feelings of loneliness – a subjective experience, indicating a general dissatisfaction with ones’ social relationships (Lodder, Scholte, Goossens, & Verhagen, 2017) – which could also predict NSSI. Accordingly, prior work revealed that loneliness is associated with higher levels of NSSI engagement (e.g. Wang & Liu, 2019), although evidence from longitudinal studies is lacking. In sum, because peer problems can be highly stressful in adolescence, they may pose risk for subsequent NSSI engagement. Yet, although existing research provided support for this hypothesis, relatively few longitudinal studies have examined the extent to which peer problems may contribute to the development and maintenance of NSSI throughout adolescence.

### NSSI as antecedent of peer problems

According to interpersonal models of developmental psychopathology (Rudolph et al., 2016), the dynamic and reciprocal exchanges between individuals’ own characteristics and the ones of their environments shape the course of development. These transactional models posit that individuals are not simply passive receivers of experiences but they actively contribute to their social contexts with certain attitudes and behaviours that in some cases may further increase their (interpersonal) stress levels (Hammen, 1991). Thus, engaging in NSSI may have consequences for adolescents’ social relationships, including the ones with their peers.

Although NSSI engagement may be driven by (perceived) interpersonal benefits (e.g. facilitate help‐seeking), these behaviours may also elicit negative reactions from others (You, Leung, Lai, & Fu, 2012). Recent work has revealed that NSSI is often a stigmatized behaviour which could have negative consequences for mental health as well as poor social relationships (e.g. Piccirillo, Burke, Moore‐Berg, Alloy, & Heimberg, 2020). Specifically, NSSI could become a trigger for relationship problems as it may be disapproved and perceived as deviant, leading to avoidance, isolation or even rejection (You et al., 2012). Thus, adolescents who engage in NSSI may be at higher risk for experiencing subsequent peer problems.

First, adolescents who self‐injure may be more likely to be victimized, perhaps because they are perceived as different, more vulnerable, and are viewed with prejudice by their peers. This hypothesis is consistent with interpersonal scar models (or symptom‐driven models) showing that greater levels of internalizing distress are often antecedents of relationships difficulties, including peer victimization (e.g. Rudolph, 2017). Second, consistent with stress generation models (e.g. Hammen, 1991), adolescents who self‐injury may contribute to create a stressful environment, for example, within their friendships. Accordingly, a few studies revealed that among college students and adolescents, especially girls, NSSI predicted higher levels of interpersonal stress over time (e.g. Burke, Hamilton, Abramson, & Alloy, 2015; Ewing, Hamza, & Willoughby, 2019).

Finally, learning that a friend engages in NSSI also may result in negative reactions, perhaps due to stigma, prejudice or lack of understanding, which consequently could lead to social distancing and deceases in perceived social support (Hasking, Rees, Martin, & Quigley, 2015). For example, a recent review (Simone & Hamza, 2020) highlighted the possible negative impact of disclosing NSSI, which was often associated with loss of peers, threaten to end relationships and eventually subsequent increases in NSSI. Even if adolescents do not disclose NSSI, they may still be at risk for experiencing loneliness, perhaps because they feel different, ashamed or because they cannot share this with anyone (Gandhi, Luyckx, Goossens, Maitra, & Claes, 2018). In sum, existing work suggests that youth who engage in NSSI could be at increased risk for experiencing social relationship difficulties; yet, to date, no study investigated the extent to which NSSI may predict subsequent peer problems during adolescence, a susceptible period for both NSSI as well as peer relationship development.

### The current study

The present study aimed to investigate the reciprocal associations among three indicators of peer problems (i.e. peer victimization, friendship stress and loneliness) and NSSI using a six‐wave prospective design in which a large community sample of adolescents were followed throughout adolescence. These associations were investigated using Random Intercept Cross‐Lagged Panel Models (RI‐CLPM; Hamaker, Kuiper, & Grasman, 2015), in order to differentiate between‐person from within‐person effects. RI‐CLPMs allowed us to control for all unmeasured stable confounders that may explain the associations between peer problems and NSSI (e.g. genetic vulnerabilities), by removing the variance that is due to time‐invariant between‐person differences. In this way, the reciprocal effects between peer problems and NSSI were examined at the within‐person level and referred to intra‐personal deviations from individuals’ own expected levels, allowing us to know whether changes in adolescents’ own NSSI were related to subsequent deviations in their own peer problems, and vice versa. Notably, to date these models offer the closest possible approximation to identify “causal effects” using observational data (Lervåg, 2020).

The study hypotheses and the analytic approach were preregistered (https://osf.io/n67kp/?view_only=2eaa905205c540cd9df929d771f7fedc). Based on transactional models (Rudolph et al., 2016), we expected reciprocal longitudinal associations between peer problems and NSSI. Specifically, at the within‐person level, we expected that when adolescents experienced higher than usual peer relationship problems (i.e. higher levels of peer victimization, friendship stress and loneliness), they reported higher than usual NSSI engagement at the subsequent time point and vice versa. Moreover, we also hypothesized between‐person associations, indicating that adolescents with more peer problems also reported higher levels of NSSI engagement than their peers.

Gender differences in the (within‐person) reciprocal associations between peer problems and NSSI were also explored. Past research has shown that, as compared to boys, girls are more likely to engage in NSSI (Bresin & Schoenleber, 2015), report higher levels of interpersonal stress within close relationships (e.g. friendships), and tend to have greater sensitivity to social stress (Burke et al., 2015). Thus, NSSI and peer problems may influence each other over time more strongly for adolescent girls than boys. Finally, in sensitivity analyses, we explored whether the reciprocal associations between peer problems and NSSI held while accounting for within‐person fluctuations in depressive symptoms, given that high levels of depressive symptoms have been found to predict both peer problems (e.g. Rudolph, 2017) and NSSI (Fox et al., 2015).

## Methods

### Participants and procedures

Participants were 866 adolescents (54.5% females; *M*_age_ = 13.12 years at baseline, *SD* = 0.78) part of two cohorts (53.9% in Grade 7 and 46.1% in Grade 8 at baseline) and attended three rural, low‐income middle schools in a single county in south‐eastern United States. The sample was ethnically diverse, with 47.2% of adolescents identifying themselves as Caucasian, 23.1% as Latinx, 22.1% as African‐American and 7.6% as belonging to other ethnic minority groups. Based on census trac data of street addresses, participants’ household income was in the lower middle class range (*M* = $40,759.59; *SD* = $15,491.39) (www.census.gov).

All seventh‐ and eighth‐grade students in regular classrooms (*n* = 1,463) were invited to participate in the study and 59.20% of them took part in the baseline assessment (*n* = 866). Subsequently, participants were followed through high school, with assessments occurring at a year interval. Thus, in total, students in the younger cohort (i.e. Grade 7 at baseline) participated in a maximum of six waves of data collection, while students in the older cohort (i.e. Grade 8 at baseline) in five. Retention rates between consecutive assessments ranged between 88.7% and 97.7% (67% between Time 1 and Time 6). All participants with available data at one time point at least were included in the analyses (see Appendix S1). The study received ethical approval from the relevant University’s Institutional Review Board.

#### Measures

##### Non‐suicidal self‐injury (NSSI)

At each time point, NSSI was measured with six items, each rated on a 5‐point Likert scale from 1 (‘*never’*) to 5 (‘*10+times’* ), with a possible total score ranging from 6 to 30 (Prinstein et al., 2008). The scale assessed how frequently, during the past year, adolescents had engaged in six different types of non‐suicidal self‐injurious behaviour (e.g. cutting/carving, burning, hitting), without suicidal intent. A total NSSI score was calculated by summing the six items (Cronbach’s αs .79–.86).

##### Friendship stress

At each time point, friendship stress was assessed using a self‐report measure developed based on standardized questions from the Youth Life Stress Interview (YLSI; Rudolph & Flynn, 2007). The scale included 11 items (e.g. “A friend talked behind your back”) asking about common stressful events that adolescents may have experienced during the past year in the context of their friendships. Each item was rated on a 5‐point Likert‐type scale from 1 (‘*never’*) to 5 (‘*very often’*). Responses to the 11 items were averaged (Cronbach’s αs .92–.93).

##### Loneliness

From Time 2, loneliness was assessed by asking participants to rate five items on a 5‐point Likert scale ranging from 1 (‘*never*’) to 5 (‘*very often*’). Three items were an adaptation of the Loneliness and Social Dissatisfaction Questionnaire (Cassidy & Asher, 1992) (i.e. I felt alone; I felt left out of things; I was lonely) and two items were previously developed by Ladd and Burgess (1999) (i.e. School was a lonely place for me; I was sad and alone). Answers to all items were averaged to obtain an overall measure of loneliness (Cronbach’s αs .94–.95; see also Appendix S2). Notably, although most items included in this scale did not directly tapped into peer‐related loneliness, research demonstrated that generic measures of loneliness reflect loneliness within the peer context (see Goossens et al., 2009).

##### Peer victimization

From Time 2, participants took part in a peer nomination procedure to assess both overt and relational victimization (Helms et al., 2015; see Supporting Information, Appendix S2). Students were asked to nominate an unlimited number of peers within their grade, in response to the following questions: “Who gets threatened or physically hurt by others?” (i.e. overt victimization) and “Who gets left out of activities, ignored by others because one of their friends is mad at them, gossiped about, or has mean things said behind their backs?” (i.e. relational victimization). For each student, the total number of nominations received were summed and standardized within grade for both the overt and relational victimization item separately. At Time 2, participants received on average 1.30 and 1.86 (*SDs* = 2.70 and 2.89) nominations for overt and relational victimization respectively. Subsequently, given the moderate to strong correlation between the items (*rs* = .42–.82 across waves) a total peer victimization score was computed by averaging across the standardized scores of relational and overt victimization. Because peer victimization was highly skewed, before analyses a log_10_ transformation was applied and extreme outliers (i.e. values higher than 3*SD* above the mean) were winsorized to the highest value in the distribution within 3 *SD*s from the mean (Grade 8, *n* = 9; Grade 9, *n* = 12; Grade 10, *n = *7; Grade 11, *n* = 8 and Grade 12, *n* = 6).

##### Depressive symptoms

At each time point, depressive symptoms were assessed using the Short Mood and Feeling Questionnaire (SMFQ; Angold et al., 1995). This scale includes 13 items (e.g. “I felt miserable or unhappy”), describing depressive symptoms that participants may have experienced during the previous 2 weeks. Each item was rated on a 3‐point Likert scale from 0 (*‘not true’*) to 2 (*‘true’*). Reponses to the 13 items were averaged (Cronbach’s αs .92–.95).

### Plan of analysis

Analyses were carried out consistent with our preregistration (see, https://osf.io/n67kp/?view_only=2eaa905205c540cd9df929d771f7fedc) unless differently indicated. To examine the bi‐directional associations between peer problems and NSSI, a series of RI‐CLPMs (Hamaker et al., 2015) was estimated. In these models, the variance of the observed variables is decomposed into stable latent factors that are invariant over time, reflecting between‐person (i.e. trait‐like) differences, and multiple time‐variant latent factors reflecting within‐person deviations from the person’s own expected score (Hamaker et al., 2015). Separate models were fitted for each of the three peer problems, that is, peer victimization, friendship stress and loneliness (see Appendix S3).

A series of supplementary analyses was also conducted. First, the main study associations were examined using traditional Cross‐lagged Panel Models (CLPMs), to explore whether model fits improved using RI‐CLPM. Second, gender differences were explored using a multi‐group approach, in which models with all paths freely estimated across gender were compared to models in which the cross‐lagged paths were fixed to be equal across gender. Finally, as sensitivity analyses (i.e. not pre‐registered), we estimated RI‐CLPMs including bi‐directional associations between peer problems, NSSI as well as depression, to explore the robustness of the findings when controlling for within‐person fluctuations in depression symptoms. All main analyses were conducted in Mplus version 7 (Muthen & Muthen, 2012).

## Results

### Descriptive analysis

Across the six assessments, between 17.3% and 31.9% of adolescents reported that they had engaged in at least one NSSI episode during the previous year (i.e. T1 = 32.1%, T2 = 30.1%, T3 = 27.2%, T4 = 24.7%, T5 = 21.1%, T6 = 17.3%). The bivariate correlations between peer problems and NSSI are reported in Table S1. Intraclass correlations (ICCs) ranged between .41 (for NSSI) and .56 (for loneliness), suggesting that between 41% and 56% of the observed variance in the main study variables was due to stable between‐person differences, while the remaining variance was attributable to within‐person fluctuations over time.

### Reciprocal associations between peer problems and NSSI

**Figure 1:**
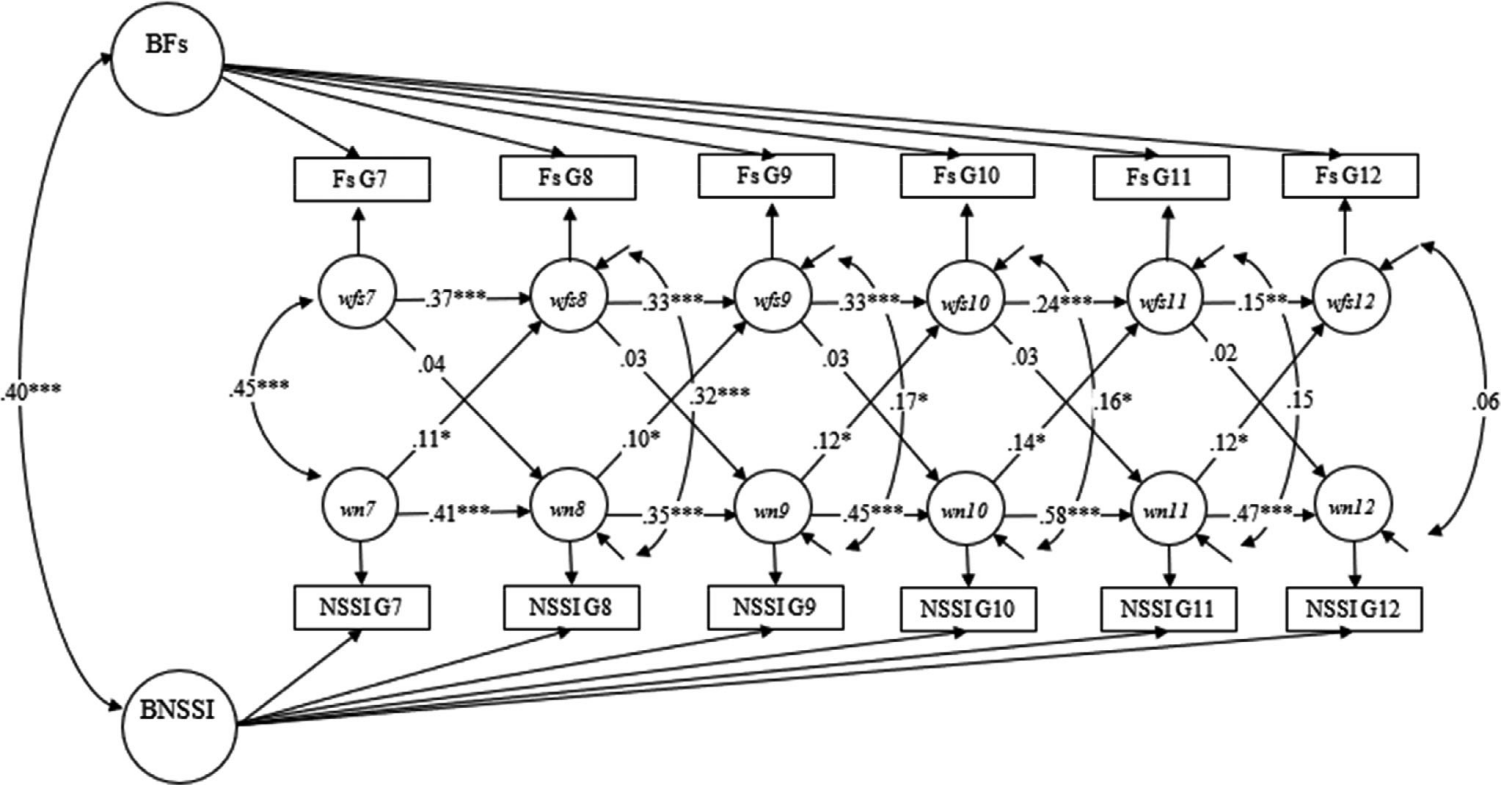
Final random intercept cross‐lagged panel model between friendship stress and NSSI. Note. BFs = Between‐person random intercept for friendship stress; BNSSI = Between‐person random intercept for NSSI; FsG7‐FsG12 = observed friendship stress scores from Grade 7 to 12; NSSIG7‐NSSIG12 = observed NSSI scores from Grade 7 to 12; *wfs7‐wfs12* = within‐person latent factors for friendship stress from Grade 7 to 12; *wn7‐wn12* = within‐person latent factors for NSSI from Grade 7 to 12. Standardized estimates are reported. Model fit: *χ* ^2^ (45) = 47.187 (*p* = .383), RMSEA = .007 (90% CI = [.000,.024]), CFI = 0.999, TLI = 0.998 and WRMR = 0.525. *p < .05; **p < .01; ***p < .001

Model comparisons conducted to test time invariance of the estimates are reported and described in the Supporting Information (see also Appendices S4–S6; Tables S2–S4). Figure 1 (see also Figures S1 and S2) displays the final RI‐CLPMs (see also Tables S5–S7 for *SEs* and 95%CI; Appendices S7–S9), including model fits. All RI‐CLPMs fitted the data significantly better than traditional CLPMs, indicating the need to distinguish between‐ and within‐person effects (Appendix S10; Table S8; Figures S3–S5).

At the between‐person level, more friendship stress and loneliness, but not peer victimization, were positively associated with more NSSI, with medium effect sizes. Thus, across waves, adolescents who reported higher levels of friendship stress and loneliness, but not peer victimization, also reported to engage in NSSI more often. Positive and significant (within‐person) cross‐lagged effects from NSSI to all three types of peer problems were also revealed. These effects were small in size and indicated that when adolescents reported higher levels of NSSI (as compared to their own mean) they also reported higher levels of friendship stress, loneliness and peer victimization (as compared to their own mean) at the subsequent time point. However, no reverse effects from peer problems to NSSI were found. Furthermore, comparing the strength of the cross‐lagged effects from peer problems to NSSI with the ones from NSSI to peer problems, using model comparisons, generally resulted in worst model fits, Δχ^2^ (1) = 4.859, *p* = .027 (for friendship stress), Δχ2 (1) = 9.792, *p* = .002 (for loneliness) and Δχ^2^ (1) = 3.699, *p* = .054 (for peer victimization). This suggests that the effects from NSSI to peer problems were overall stronger than the effects from peer problems to NSSI.

### Supplementary analyses

All RI‐CLPMs estimated to explore gender differences (Appendix S11; Table S9) and the confounding effects of depressive symptoms (Appendices S12–S14; Tables S10–S12; Figures S6–S8) are reported in the Supporting Information. Constraining the cross‐lagged effects to be equal across gender did not worsen the model fits (see Table S9), indicating that the within‐person reciprocal effects between peer problems and NSSI were similar for boys and girls. In the RI‐CLPMs including depressive symptoms, the between‐person associations between peer problems and NSSI remained unchanged. However, the within‐person effects of NSSI on peer problems were strongly attenuated, so that they only reached significance for peer victimization (from Grade 8 to 9, and marginal significance from Grade 10 to 11), and approached significance for friendship stress (see Figures S6–S8). Interestingly, NSSI positively predicted within‐person changes in depression, but not vice versa; besides, positive reciprocal within‐person cross‐lagged effects were found between depressive symptoms and both friendship stress and loneliness.

## Discussion

Despite evidence that adolescents who experience peer problems are at greater risk for engaging in NSSI, little is known about the possible consequences of NSSI for adolescents’ relationships with their peers. This study contributed to this research by examining, for the first time, how peer problems (i.e. peer victimization, friendship stress and loneliness) and NSSI may reciprocally affect one another throughout adolescence, using a state‐of‐the‐art analytic technique that allowed us to differentiate between‐person from within‐person effects. Findings revealed that loneliness and friendship stress, but not peer victimization, were associated with NSSI primarily at the between‐person level, indicating that adolescents who reported more loneliness and friendship stress also reported more NSSI engagement. After controlling for these between‐person associations, adolescents’ higher‐than‐usual levels of NSSI were also found to predict subsequent higher‐than‐usual levels in their own peer problems, although sensitivity analyses revealed that these effects were mostly explained by within‐person fluctuations in depressive symptoms. These findings advance our current understanding of adolescent NSSI and have a number of noteworthy theoretical and practical implications.

Consistent with our hypothesis, the between‐person associations between NSSI and both loneliness and friendship stress supported the possibility that NSSI and difficulties with peers may, in part, be manifestations of stable, shared underlying vulnerabilities. For example, personality traits, such as neuroticism, or genetic predispositions may increase the risk for both engaging in NSSI and experiencing higher levels of loneliness and friendship stress (e.g. Kiekens et al., 2015). However, the lack of between‐person association between NSSI and peer victimization was unexpected. This finding may, in part, stem from the different assessment method used to measure NSSI (i.e. self‐report) and peer victimization (i.e. peer nomination procedure), and it indicates that those adolescents who had a ‘victim’ reputation among their peers were not necessarily those who also engaged more often in NSSI. At the within‐person level, although we hypothesized bi‐directional effects over time, initial results indicated that NSSI consistently increased the risk for all type of peer problems, but not vice versa, similarly among boys and girls. These findings extend prior work on NSSI as predictor of interpersonal stress over time (e.g. Burke et al., 2015; Ewing et al., 2019), and consistent with interpersonal theories of developmental psychopathology (Rudolph et al., 2016) suggest that adolescents who engage in NSSI may shape their social environment in a way that could potentially deprive them from a positive social context fundamental for their development.

Yet, these findings should also be interpreted in light of the sensitivity analyses which included depressive symptoms. First, in these analyses, NSSI no longer predicted loneliness over time, yet bi‐directional relationships between depressive symptoms and loneliness emerged. While these results may indicate that the effects of NSSI on loneliness were spurious, it is also plausible that higher levels of depressive symptoms that predicted higher loneliness over time were in part explained by co‐occurring more frequent NSSI engagement (as evident in the concurrent within‐person association between depression and NSSI). Second, after controlling for depression, NSSI only marginally predicted friendship stress over time, yet it was positively associated with subsequent depressive symptoms, which in turn were associated with higher‐than‐usual levels of friendship stress at the following time point. These findings are remarkable as they imply possible cascade effects from NSSI to stress exposure within friendships. Specifically, NSSI can be a cue of negative emotions and depressive feelings that, as suggested by the stress generation hypothesis (Hammen, 1991), may in turn contribute actively to create a stressful environment. Thus, depression may represent a mechanism through which NSSI poses risk for stressful experiences within friendship, similar to emotion dysregulation, as reported in prior work (e.g. Ewing et al., 2019), as well as externalizing problems. Indeed, research has shown that adolescents who report higher levels of externalizing problems (e.g. aggression, irritability), which often co‐occur with NSSI engagement (e.g. Tang et al., 2013), are at increased risk for experiencing peer problems (e.g. peer rejection; see Prinstein & Giletta, 2016).

Finally, in the models with peer victimization, the effects of NSSI on depressive symptoms as well as peer victimization were replicated, although the latter not consistently over time. Because in these models, depression did not predict peer victimization, high levels of depressive symptoms are unlikely to underlie the link between NSSI and peer victimization. Instead, a possible explanation for these findings could be that adolescents who self‐injure may be stigmatized and perceived as deviant, which could increase their risk for being rejected and victimized by their peers (e.g. Piccirillo et al., 2020). In sum, altogether these findings provide some preliminary support that NSSI may increase the risk for being victimized and, via elevations in depressive symptoms, for experiencing more stress within friendships. However, future research is needed to replicate the effects of depressive symptoms (which were not preregistered) as well as to directly examine the role of externalizing symptoms as possible mechanisms linking NSSI to peer problems.

Differently from what we expected, no evidence of peer problems as predictors of subsequent engagement in NSSI emerged in any model. This finding is in contrast with prior work, according to which difficulties with peers can lead to higher levels of NSSI (e.g. Giletta et al., 2015; You et al., 2012). At least two main reasons could explain these discrepant results. First, prior studies investigated the effects of peer problems on NSSI using traditional analytic techniques that confound between‐ and within‐person effects (e.g. cross‐lagged panel models; You et al., 2012). Although in our study, results from the RI‐CLPMs and the CLPMs were rather consistent, prior work indicated that not disentangling between‐person from within‐person effects may yield very different results (Nelemans et al., 2020). Second, the time span between consecutive assessments may have been too long to capture the possible effects of peer problems on NSSI. In fact, stressful peer relationships may be temporally delimited or perhaps may constitute a more proximal trigger for the engagement in NSSI (e.g. Liu et al., 2016). Specifically, NSSI may be a maladaptive way to regulate stressful situations in the short run, yet these effects are more likely observable sooner rather than 1 year later. Similarly, it is plausible that the effects of NSSI on peer problems could also be accentuated or change within shorten time periods. For example, consistent with theoretical works on the functions of NSSI, NSSI engagement may contribute to short‐term social benefits (e.g. increased support) that, however, could be only observed when examining changes over days or perhaps even hours. Yet, in the longer run – over the course of 1 year – NSSI may be more likely to undermine adolescents’ social and mental health. These hypotheses require to be directly examined in future studies.

Concerning practical implications, findings suggest the importance to promote both school environment focused strategies as well as individual‐level strategies. These could support youth in seeking care for reducing NSSI urges and behaviour, as well as emotional reactions and environmental stressors that can increase the risk for NSSI. For example, universal, school‐based programs, such as the Youth Aware of Mental Health (YAM), could be beneficial in reducing stigma, through mental health awareness, and in increasing overall support as well as youth emotion regulation and problem‐solving skills (Lindow et al., 2020).

Besides, our findings suggest that clinical intervention should also attend to support adolescents who engage in NSSI (i.e. individual prevention) in order to prevent the development and maintenance of internalizing symptoms (e.g. depression) and possible peer difficulties, for example, by targeting emotion regulation skills.

This study has a number of strengths, including the large sample, the multi‐wave design and the analytic approach (i.e. RI‐CLPM) that differentiates between‐person and within‐person associations. Notably, this approach allowed us to examine the extent to which peer problems and NSSI reciprocally influenced each other over time, while taking into account all stable factors that may have influenced both peer problems and NSSI. Despite these strengths, the current results should be considered in light of some limitations. First, limitations related to the study measures should be noted. The self‐report assessment of NSSI may have been affected by social desirability, respondent and recall bias, or may have been subject to possible misinterpretation (e.g. in the definition of NSSI). Moreover, the measure used to assess loneliness was not validated and did not directly ask about peer‐related loneliness. Thus, these findings should be replicated, for instance, using clinical interviews or multi‐method assessments of NSSI.

Second, although RI‐CLPMs to date offer one of the most suitable analytic approaches to strengthen causal inferences from observational data, the use of a non‐experimental design still does not allow us to draw causal conclusions. Moreover, due to the complexity of these models and the large sample sizes required to identify small effects, we decided to examine associations between NSSI and each of the three peer problems in separate models. Thus, it remains unclear whether NSSI may simultaneously pose risk for different types of peer problems, or whether some of the observed associations are in fact redundant. Third, additional contextual factors, such as school‐level characteristics, were not considered. Previous work has shown that rates of self‐injurious behaviours may be affected by school‐level factors, such as peer network cohesion in school (Wyman et al., 2019); therefore, future work is warranted to examine the extent to which these broader level school factors may also influence the associations between peer problems and NSSI. Finally, as discussed above, the time span between waves was 12 months, and therefore it may have been too long to identify within‐person effects (Dormann & Griffin, 2015).

In conclusion, the present study offers the first in‐depth investigation on how NSSI and different types of peer problems may reciprocally influence each other over the course of adolescence. Findings highlight that the links between NSSI and both loneliness and friendship stress may be due to stable, common underlying factors. However, some evidence also indicates that NSSI engagement may increase adolescents’ vulnerability to be exposed to peer victimization as well as stress within their friendships, likely because NSSI puts them at risk for experiencing higher levels of depressive symptoms. Altogether, these findings underscore the need to pay attention to the possible mental and social risks of NSSI engagement during adolescence.

## Supporting information


**Appendix S1**. Study Procedures.
**Appendix S2**. Study Measures.
**Appendix S3**. Plan Analysis.
**Table S1**. Bivariate Correlations between Peer Problems and Non‐Suicidal Self‐Injury across all Time Points.
**Appendix S4**. Random Intercept Cross‐Lagged Panel Model (RI‐CLPM) between Friendship Stress and Non‐Suicidal Self‐Injury.
**Table S2**. Model Fit Indices and Model Fit Comparisons of Random‐Intercept Cross‐lagged Panel Models between Friendships Stress and Non‐Suicidal Self‐Injury.
**Appendix S5**. Random Intercept Cross‐Lagged Panel Model (RI‐CLPM) between Loneliness and Non‐Suicidal Self‐Injury.
**Table S3**. Model Fit Indices and Model Fit Comparisons of Random‐Intercept Cross‐lagged Panel Models between Loneliness and Non‐Suicidal Self‐Injury.
**Appendix S6**. Random Intercept Cross‐Lagged Panel Model (RI‐CLPM) between Peer Victimization and Non‐Suicidal Self‐Injury.
**Table S4**. Model Fit Indices and Model Fit Comparisons of Random‐Intercept Cross‐lagged Panel Models between Peer Victimization and Non‐Suicidal Self‐Injury.
**Table S5**. Within‐Person Effects for the Final Model (Model 7) between Friendship Stress and Non‐Suicidal Self‐Injury.
**Appendix S7**. Reciprocal Associations between Friendship Stress and NSSI at the Within‐Person Level.
**Table S6**. Within‐Person Effects for the Final Model (Model 7) between Loneliness and Non‐Suicidal Self‐Injury.
**Figure S1**. Final Random‐Intercept Cross‐lagged Panel Model between Loneliness and NSSI.
**Appendix S8**. Reciprocal Associations between Loneliness and NSSI at the Within‐Person Level.
**Table S7**. Within‐Person Effects for the Final Model (Model 7) between Peer Victimization and Non‐Suicidal Self‐Injury.
**Figure S2**. Final Random‐Intercept Cross‐lagged Panel Model between Peer Victimization and NSSI.
**Appendix S9**. Reciprocal Associations between Peer Victimization and NSSI at the Within‐Person Level.
**Appendix S10**. Cross‐lagged Panel Models (CLPM) between Peer Problems and Non‐Suicidal Self‐Injury.
**Table S8**. Model Fit Indices and Model Fit Comparisons of Cross‐lagged Panel Models between Peer Problems and Non‐Suicidal Self‐Injury.
**Figure S3**. Diagram of Final Cross‐Lagged Panel Model between Friendship Stress and Non‐Suicidal Self‐Injury.
**Figure S4**. Diagram of Final Cross‐Lagged Panel Models between Loneliness and Non‐Suicidal Self‐Injury.
**Figure S5**. Diagram of Final Cross‐Lagged Panel Models between Peer‐Victimization and Non‐Suicidal Self‐Injury.
**Appendix S11**. Gender Moderation Analysis.
**Table S9**. Model Fit Indices and Model Fit Comparisons for Gender Multi‐Group Random‐Intercept Cross‐lagged Panel Models.
**Appendix S12**. Random Intercept Cross‐Lagged Panel Model (RI‐CLPM) with Friendship Stress, Depression and NSSI.
**Table S10**. Model Fit Indices and Model Fit Comparisons of Random‐Intercept Cross‐Lagged Panel Models with Friendships Stress, Depression and Non‐Suicidal Self‐Injury.
**Figure S6**. Final Between‐ and Within‐Person Effect of the Random‐Intercept Cross‐lagged Panel Model with Friendship Stress, Depression and NSSI.
**Appendix S13**. Random Intercept Cross‐Lagged Panel Model (RI‐CLPM) with Loneliness, Depression and NSSI.
**Table S11**. Model Fit Indices and Model Fit Comparisons of Random‐Intercept Cross‐Lagged Panel Models with Loneliness, Depression and Non‐Suicidal Self‐Injury.
**Figure S7**. Final Between‐ and Within‐Person Effect of the Random‐Intercept Cross‐lagged Panel Model with Loneliness, Depression and NSSI.
**Appendix S14**. Random Intercept Cross‐Lagged Panel Model (RI‐CLPM) with Peer Victimization, Depression and NSSI.
**Table S12**. Model Fit Indices and Model Fit Comparisons of Random‐Intercept Cross‐Lagged Panel Models with Peer Victimization, Depression and Non‐Suicidal Self‐Injury.
**Figure S8**. Final Between‐ and Within‐Person Effect of the Random‐Intercept Cross‐lagged Panel Model with Peer Victimization, Depression and NSSI.
