# Supplementary material for: Reciprocal associations between peer problems and non‐suicidal self‐injury throughout adolescence
Source: J Child Psychol Psychiatry. 2022 Apr 5;63(12):1486–95. doi: 10.1111/jcpp.13601 (PMC9790606; doi:10.1111/jcpp.13601)
Supplement: Supplementary file 1 — Appendix S1. Study Procedures. Appendix S2. Study Measures. Appendix S3. Plan Analysis. Table S1. Bivariate Correlations between Peer Problems and Non‐Suicidal Self‐Injury across all Time Points. Appendix S4. Random Intercept Cross‐Lagged Panel Model (RI‐CLPM) between Friendship Stress and Non‐Suicidal Self‐Injury. Table S2. Model Fit Indices and Model Fit Comparisons of Random‐Intercept Cross‐lagged Panel Models between Friendships Stress and Non‐Suicidal Self‐Injury. Appendix S5. Random Intercept Cross‐Lagged Panel Model (RI‐CLPM) between Loneliness and Non‐Suicidal Self‐Injury. Table S3. Model Fit Indices and Model Fit Comparisons of Random‐Intercept Cross‐lagged Panel Models between Loneliness and Non‐Suicidal Self‐Injury. Appendix S6. Random Intercept Cross‐Lagged Panel Model (RI‐CLPM) between Peer Victimization and Non‐Suicidal Self‐Injury. Table S4. Model Fit Indices and Model Fit Comparisons of Random‐Intercept Cross‐lagged Panel Models between Peer Victimization and Non‐Suicidal Self‐Injury. Table S5. Within‐Person Effects for the Final Model (Model 7) between Friendship Stress and Non‐Suicidal Self‐Injury. Appendix S7. Reciprocal Associations between Friendship Stress and NSSI at the Within‐Person Level. Table S6. Within‐Person Effects for the Final Model (Model 7) between Loneliness and Non‐Suicidal Self‐Injury. Figure S1. Final Random‐Intercept Cross‐lagged Panel Model between Loneliness and NSSI. Appendix S8. Reciprocal Associations between Loneliness and NSSI at the Within‐Person Level. Table S7. Within‐Person Effects for the Final Model (Model 7) between Peer Victimization and Non‐Suicidal Self‐Injury. Figure S2. Final Random‐Intercept Cross‐lagged Panel Model between Peer Victimization and NSSI. Appendix S9. Reciprocal Associations between Peer Victimization and NSSI at the Within‐Person Level. Appendix S10. Cross‐lagged Panel Models (CLPM) between Peer Problems and Non‐Suicidal Self‐Injury. Table S8. Model Fit Indices and Model Fit Comparisons of Cr [file JCPP-63-1486-s001.docx]

PEER PROBLEMS AND NSSI IN ADOLESCENCE

Supporting information for **Reciprocal Associations between Peer Problems and Non-Suicidal Self-Injury throughout Adolescence**

- **Appendix S1.** Study Procedures
- **Appendix S2.** Study Measures
- **Appendix S3.** Plan Analysis
- **Table S1.** Bivariate Correlations between Peer Problems and Non-Suicidal Self-Injury across all Time Points
- **Appendix S4.** Random Intercept Cross-Lagged Panel Model (RI-CLPM) between Friendship Stress and Non-Suicidal Self-Injury
- **Table S2.** Model Fit Indices and Model Fit Comparisons of Random-Intercept Cross-lagged Panel Models between Friendships Stress and Non-Suicidal Self-Injury
- **Appendix S5.** Random Intercept Cross-Lagged Panel Model (RI-CLPM) between Loneliness and Non-Suicidal Self-Injury
- **Table S3.** Model Fit Indices and Model Fit Comparisons of Random-Intercept Cross-lagged Panel Models between Loneliness and Non-Suicidal Self-Injury
- **Appendix S6.** Random Intercept Cross-Lagged Panel Model (RI-CLPM) between Peer Victimization and Non-Suicidal Self-Injury
- **Table S4.** Model Fit Indices and Model Fit Comparisons of Random-Intercept Cross-lagged Panel Models between Peer Victimization and Non-Suicidal Self-Injury
- **Table S5.** Within-Person Effects for the Final Model (Model 7) between Friendship Stress and Non-Suicidal Self-Injury
- **Appendix S7.** Reciprocal Associations between Friendship Stress and NSSI at the Within-Person Level
- **Table S6.** Within-Person Effects for the Final Model (Model 7) between Loneliness and Non-Suicidal Self-Injury
- **Figure S1.** Final Random-Intercept Cross-lagged Panel Model between Loneliness and NSSI
- **Appendix S8.** Reciprocal Associations between Loneliness and NSSI at the Within-Person Level
- **Table S7.** Within-Person Effects for the Final Model (Model 7) between Peer Victimization and Non-Suicidal Self-Injury
- **Figure S2.** Final Random-Intercept Cross-lagged Panel Model between Peer Victimization and NSSI
- **Appendix S9.** Reciprocal Associations between Peer Victimization and NSSI at the Within-Person Level
- **Appendix S10.** Cross-lagged Panel Models (CLPM) between Peer Problems and Non-Suicidal Self-Injury
- **Table S8.** Model Fit Indices and Model Fit Comparisons of Cross-lagged Panel Models between Peer Problems and Non-Suicidal Self-Injury
- **Figure S3.** Diagram of Final Cross-Lagged Panel Model between Friendship Stress and Non-Suicidal Self-Injury
- **Figure S4.** Diagram of Final Cross-Lagged Panel Models between Loneliness and Non-Suicidal Self-Injury
- **Figure S5.** Diagram of Final Cross-Lagged Panel Models between Peer-Victimization and Non-Suicidal Self-Injury
- **Appendix S11.** Gender Moderation Analysis
- **Table S9.** Model Fit Indices and Model Fit Comparisons for Gender Multi-Group Random-Intercept Cross-lagged Panel Models
- **Appendix S12.** Random Intercept Cross-Lagged Panel Model (RI-CLPM) with Friendship Stress, Depression and NSSI
- **Table S10.** Model Fit Indices and Model Fit Comparisons of Random-Intercept Cross-Lagged Panel Models with Friendships Stress, Depression and Non-Suicidal Self-Injury
- **Figure S6.** Final Between- and Within-Person Effect of the Random-Intercept Cross-lagged Panel Model with Friendship Stress, Depression and NSSI.
- **Appendix S13.** Random Intercept Cross-Lagged Panel Model (RI-CLPM) with Loneliness, Depression and NSSI.
- **Table S11.** Model Fit Indices and Model Fit Comparisons of Random-Intercept Cross-Lagged Panel Models with Loneliness, Depression and Non-Suicidal Self-Injury.
- **Figure S7.** Final Between- and Within-Person Effect of the Random-Intercept Cross-lagged Panel Model with Loneliness, Depression and NSSI.
- **Appendix S14.** Random Intercept Cross-Lagged Panel Model (RI-CLPM) with Peer Victimization, Depression and NSSI.
- **Table S12.** Model Fit Indices and Model Fit Comparisons of Random-Intercept Cross-Lagged Panel Models with Peer Victimization, Depression and Non-Suicidal Self-Injury
- **Figure S8.** Final Between- and Within-Person Effect of the Random-Intercept Cross-lagged Panel Model with Peer Victimization, Depression and NSSI.

**Appendix S1. Study Procedures**

Below, additional information on the study procedures is reported (e.g., administration of questionnaires; informed consent; study attrition).

Consent forms were distributed to all students’ families and 90% of those were returned, with 74% of the parents giving consent for their child to participate in the study. The vast majority of adolescents with parental consent took part in the data collection at baseline (*n* = 866; 59.20% of the targeted population). Study attrition was due to participants’ moving from the area, absenteeism, incomplete data, and declining to continue participation. To compare participants with and without missing data, Little’s (1988) Missing Completely at Random (MCAR) tests were performed. Because the associations between NSSI and each of the peer problems variable were examined in separate models (see Analysis plan), three different tests were conducted. Although all tests emerged to be significant (friendship stress: χ^2^ (449) =536.071, *p*=.003; loneliness: χ2 (297) =363.266, *p*=.005; peer victimization: χ2 (403) =628.442, *p*<.001), the normed χ^2^/df, ranging between 1.19 and 1.56, suggest that data were likely missing at random (Bollen, 1989). Questionnaires were administered at school following the same procedures at all time points, using computer-assisted self-interviewing (CASI) with dividers between students to ensure privacy. Participants were compensated with a $10 gift card at each assessment.

**Appendix S2. Study Measure**

*Loneliness.* To measure loneliness, a combination of items from previously developed scales was used. Therefore, first, to examine whether all the selected items tapped into one common factor, a Confirmatory Factor Analysis (CFA) was conducted at each time point. Consistently across time points, results from the CFA showed a good model fit (e.g., Model fit for Time 2 – when loneliness was assessed for the first time – χ^2^(5) = 26.550, *p*<.001 CFI = 0.972; TLI=.944, RMSEA = 0.076, 90% CI [0.145, 0.199], SRMR=0.017), supporting the unidimensional nature of this measure. Moreover, in support of the validity of this measure, loneliness was associated with related constructs, including depressive symptoms (see Table S1) and self-esteem (*r*= -.40) – which was also assessed with a single-item measure in the larger project at Time 4 – as well as peer-related constructs (i.e., peer victimization and friendship stress; see Table S1). Notably, these correlations are consistent and comparable in size to the ones emerged in prior work using other loneliness scales (e.g., Lasgaard, 2007; Mahon et al., 2006).

*Peer victimization.* A peer nomination procedure was used to assess peer victimization. This approach allowed us not to rely exclusively on self-reported measures, which is particularly important when examining associations with internalizing problems, given that adolescents with higher levels of internalizing problems may tend to have distorted views of their social environment, and overestimate negative experiences, including peer victimization (De Los Reyes & Prinstein, 2004).

It should be noted, however, that our participation rate was below the 60-70% traditionally recommended to use peer nomination procedures (Cillessen & Marks, 2011), which possibly may have impacted the reliability of this measure. However, prior work examining the reliability of peer nomination measures (Marks et al., 2013) has yielded two important findings, that provide support for the use of peer nominations in our study. First, when peer nominations are used to assess constructs that are based on observable behaviors, such as aggression (e.g., ‘who start fights, say mean things, and/or tease others’), rather than on “individual affective reactions” (Marks et al., 2013; p. 618), such as likeability and friendships, good reliabilities are observed even with participation rates as low as 40% (Marks et al., 2013). Second, peer nomination procedures that include more data, for instance by using unlimited (rather than limited) nominations, grade-wide (rather than class-wide) nominations, and that rely on more than one item, as done in our study, yield significantly higher reliability indices.

Finally, it should be noted that, especially in North America, grade-wide, rather than class-wide, approaches are typically used among middle and high school students, given that starting from early adolescence students have extensive social interactions with other peers beyond the ones in their classroom context (Cillessen & Marks, 2017). Thus, using grade, rather than classroom-wide, peer nominations are more suitable among adolescents.

**Appendix S3. Plan Analysis**

All Random Intercept Cross-Lagged Panel Models (RI-CLPMs) were estimated using grade, rather than wave of data collection, as time metric. Grade was used as time metric because this study recruited two different cohorts (Grade 7 and 8 at baseline) and data were collected over time with an interval of one year between consecutive assessments. Thus, this allowed us to examine effects over a developmental period from grade 7 to 12, with data missing by design for some participants (i.e., data at grade 7 for students entering the study in grade 8; Bollen & Curran, 2006). However, because loneliness and peer victimization were assessed only starting from Time 2, models including these variables were examined from grade 8 to 12. Because of this, the sample size was reduced to 829 and 835 participants for models including loneliness and peer victimization respectively.

Due to the non-normal distribution of NSSI, RI-CLPMs were estimated using a censored-normal distribution to account for the preponderance of zeros in the data (i.e., floor effect), with the Weighted Least Square Mean and Variance adjusted (WLSMV) estimation in Mplus. A censored-normal distribution has been previously used to model NSSI (e.g., Giletta et al., 2015), because this approach allows the treatment of NSSI as a continuous variable while accounting for the fact that many adolescents do not report any engagement in NSSI. This approach deviates from our pre-registered analyses, as we initially aimed and attempted to model NSSI using a (zero-inflated) negative binomial distribution; yet this option was not available in combination with RI-CLPMs. In our preregistration, we also reported that as an alternative to the (zero-inflated) negative binomial distribution we would have log10 transformed the NSSI variables to correct for skewness. This approach however did not reduce skewness, due to the high proportion of participants reporting no NSSI engagement. Thus, based on Muthén’s suggestion (Schultzberg et al., 2017), we opted for a censored-normal approach with WLSMV estimation. Note that the use of WLSMV estimation in Mplus – rather than maximum likelihood (ML) estimation – also resulted in additional deviations from the preregistration (e.g., chi-square test for difference testing was used for comparing models rather than Satorra–Bentler-scaled chi-square difference tests, as the latter is not appropriate for the WLSMV estimator).

To evaluate the model fit, traditional goodness-of-fit indices were used, including the chi-square (χ2) statistic, the root-mean-squared error of approximation (RMSEA), the comparative fit index (CFI) and the Tucker‐Lewis Index (TLI). Models were initially tested with all paths freely estimated over time, to allow for possible developmental differences in the examined effects; subsequently, model constraints were used to test for time invariance of effects. Models with RMSEA less than .08, CFI and TLI higher than .90 were considered to have acceptable fit; models with RMSEA less than .05, CFI and TLI higher than .95 were considered to have good fit (Bollen, 1989; Hu & Bentler, 1998; Yu, 2002).

To examine whether effects were similar over time (i.e., time-invariance of effects), we compared models with freely estimated paths to models in which paths were fixed to be equal over time. Time invariance of model estimates were examined by comparing groups of effects, starting with autoregressive effects - separately for peer problems and NSSI - followed by concurrent associations (i.e., within-time residual covariances and variances) and finally cross-lagged effects, separately for the peer problems and for NSSI. Nested models were compared using chi-square difference tests (Asparouhov et al., 2006; Mulder & Hamaker, 2021). Whenever the constrained model did not result in a significantly worse fit than the unconstrained model (i.e., non-significant chi-square difference test), the constrained model was retained. Finally, we compared the strength of the cross-lagged effects from peer problems to NSSI with the cross-lagged effects from NSSI to peer problems, to examine whether significant differences emerged in the extent to which these constructs reciprocally influenced each other over time.

**Table S1**

*Bivariate Correlations between Peer Problems and Non-Suicidal Self-Injury across all Time Points.*

|  | 1. | 2. | 3. | 4. | 5. | 6. | 7. | 8. | 9. | 10. | 11. | 12. | 13. | 14. | 15. | 16. | 17. | 16. | 19. | 20. | 21. | 22. | 23. | 24. | 25. | 26. | 27. | 28. |
| --- | --- | --- | --- | --- | --- | --- | --- | --- | --- | --- | --- | --- | --- | --- | --- | --- | --- | --- | --- | --- | --- | --- | --- | --- | --- | --- | --- | --- |
| 1. Friend stress T1 | - |  |  |  |  |  |  |  |  |  |  |  |  |  |  |  |  |  |  |  |  |  |  |  |  |  |  |  |
| 2. Friend stress T2 | .61^**^ | - |  |  |  |  |  |  |  |  |  |  |  |  |  |  |  |  |  |  |  |  |  |  |  |  |  |  |
| 3. Friend stress T3 | .48^**^ | .61^**^ | - |  |  |  |  |  |  |  |  |  |  |  |  |  |  |  |  |  |  |  |  |  |  |  |  |  |
| 4. Friend stress T4 | .42^**^ | .53^**^ | .60^**^ | - |  |  |  |  |  |  |  |  |  |  |  |  |  |  |  |  |  |  |  |  |  |  |  |  |
| 5. Friend stress T5 | .35^**^ | .42^**^ | .45^**^ | .52^**^ | - |  |  |  |  |  |  |  |  |  |  |  |  |  |  |  |  |  |  |  |  |  |  |  |
| 6. Friend stress T6 | .39^**^ | .43^**^ | .52^**^ | .54^**^ | .55^**^ | - |  |  |  |  |  |  |  |  |  |  |  |  |  |  |  |  |  |  |  |  |  |  |
| 7. Lon T2 | .43^**^ | .55^**^ | .43^**^ | .38^**^ | .27^**^ | .29^**^ | - |  |  |  |  |  |  |  |  |  |  |  |  |  |  |  |  |  |  |  |  |  |
| 8. Lon T3 | .37^**^ | .43^**^ | .50^**^ | .41^**^ | .29^**^ | .25^**^ | .67^**^ | - |  |  |  |  |  |  |  |  |  |  |  |  |  |  |  |  |  |  |  |  |
| 9. Lon T4 | .30^**^ | .34^**^ | .40^**^ | .56^**^ | .35^**^ | .43^**^ | .56^**^ | .67^**^ | - |  |  |  |  |  |  |  |  |  |  |  |  |  |  |  |  |  |  |  |
| 10. Lon T5 | .29^**^ | .32^**^ | .33^**^ | .40^**^ | .41^**^ | .35^**^ | .50^**^ | .58^**^ | .67^**^ | - |  |  |  |  |  |  |  |  |  |  |  |  |  |  |  |  |  |  |
| 11. Lon T6 | .24^**^ | .33^**^ | .41^**^ | .39^**^ | .36^**^ | .45^**^ | .41^**^ | .50^**^ | .58^**^ | .68^**^ | - |  |  |  |  |  |  |  |  |  |  |  |  |  |  |  |  |  |
| 12. Vict T2 | .02 | .02 | -.02 | .04 | .01 | -.07 | .14^**^ | .10^*^ | .11^**^ | .13^**^ | .02 | - |  |  |  |  |  |  |  |  |  |  |  |  |  |  |  |  |
| 13. Vict T3 | .03 | .04 | .05 | .05 | .05 | -.01 | .14^**^ | .15^**^ | .13^**^ | .18^**^ | .11 | .64^**^ | - |  |  |  |  |  |  |  |  |  |  |  |  |  |  |  |
| 14. Vict T4 | .03 | .03 | .05 | .09^*^ | .08^*^ | .03 | .09^*^ | .10^**^ | .12^**^ | .12^**^ | .004 | .56^**^ | .63^**^ | ­- |  |  |  |  |  |  |  |  |  |  |  |  |  |  |
| 15. Vict T5 | .06 | .01 | .01 | .06 | .03 | -.02 | .10^*^ | .07 | .12^**^ | .11^**^ | .11 | .44^**^ | .46^**^ | .45^**^ | - |  |  |  |  |  |  |  |  |  |  |  |  |  |
| 16. Vict T6 | -.04 | -.06 | -.03 | .03 | -.03 | -.08 | .07 | -.01 | .07 | .10 | .10 | .49^**^ | .42^**^ | .45^**^ | .46^**^ | - |  |  |  |  |  |  |  |  |  |  |  |  |
| 17. NSSI T1 | .30^**^ | .26^**^ | .19^**^ | .18^**^ | .18^**^ | .17^**^ | .30^**^ | .28^**^ | .29^**^ | .26^**^ | .21^**^ | .12^**^ | .16^**^ | .13^**^ | .10^**^ | .06 | - |  |  |  |  |  |  |  |  |  |  |  |
| 18. NSSI T2 | .24^**^ | .25^**^ | .20^**^ | .17^**^ | .14^**^ | .13* | .40^**^ | .36^**^ | .28^**^ | .23^**^ | .24^**^ | .12^**^ | .16^**^ | .09^*^ | .06 | .02 | .46^**^ | - |  |  |  |  |  |  |  |  |  |  |
| 19. NSSI T3 | .20^**^ | .19^**^ | .25^**^ | .22^**^ | .19^**^ | .10 | .36^**^ | .42^**^ | .37^**^ | .28^**^ | .19^**^ | .11^**^ | .14^**^ | .07 | .05 | .01 | .38^**^ | .58^**^ | - |  |  |  |  |  |  |  |  |  |
| 20. NSSI T4 | .09^*^ | .06 | .17^**^ | .22^**^ | .19^**^ | .21^**^ | .27^**^ | .31^**^ | .41^**^ | .36^**^ | .30^**^ | .14^**^ | .15^**^ | .11^**^ | .14^**^ | .04 | .36^**^ | .41^**^ | .62^**^ | - |  |  |  |  |  |  |  |  |
| 21. NSSI T5 | .10^**^ | .10^**^ | .11^**^ | .16^**^ | .21^**^ | .25^**^ | .17^**^ | .17^**^ | .28^**^ | .33^**^ | .28^**^ | .11^**^ | .08 | .07 | .10^*^ | .04 | .25^**^ | .23^**^ | .35^**^ | .48^**^ | - |  |  |  |  |  |  |  |
| 22. NSSI T6 | .003 | .06 | .07 | .09 | .09 | .13^**^ | .05 | .07 | .11 | .02 | .26^**^ | -.04 | -.01 | -.02 | -.06 | -.11 | .19^**^ | .25^**^ | .17^**^ | .28^**^ | .39^**^ | - |  |  |  |  |  |  |
| 23. Dep T1 | .53^**^ | .44^**^ | .38^**^ | .34^**^ | .29^**^ | .27^**^ | .54^**^ | .53^**^ | .46^**^ | .47^**^ | .34^**^ | .14^**^ | .14^**^ | .11^*^ | .10^*^ | .12^**^ | .44^**^ | .36^**^ | .25^**^ | .23^**^ | .17^**^ | .05 | - |  |  |  |  |  |
| 24. Dep T2 | .45^**^ | .53^**^ | .41^**^ | .37^**^ | .25^**^ | .26^**^ | .76^**^ | .64^**^ | .50^**^ | .45^**^ | .37^**^ | .12^**^ | .09^*^ | .06 | .06 | .06 | .34^**^ | .43^**^ | .33^**^ | .22^**^ | .19^**^ | .06 | .61^**^ | - |  |  |  |  |
| 25. Dep T3 | .36^**^ | .40^**^ | .46^**^ | .39^**^ | .31^**^ | .22^**^ | .54^**^ | .79^**^ | .57^**^ | .54^**^ | .43^**^ | .09^*^ | .15^**^ | .10^*^ | .06 | .02 | .30^**^ | .32^**^ | .43^**^ | .30^**^ | .18^**^ | .04 | .55^**^ | .60^**^ | - |  |  |  |
| 26. Dep T4 | .29^**^ | .34^**^ | .40^**^ | .50^**^ | .39^**^ | .31^**^ | .47^**^ | .60^**^ | .76^**^ | .60^**^ | .49^**^ | .12^**^ | .14^**^ | .11^**^ | .14^**^ | .03 | .26^**^ | .25^**^ | .34^**^ | .42^**^ | .30^**^ | .09 | .48^**^ | .50 | .63 | - |  |  |
| 27. Dep T5 | .27^**^ | .27^**^ | .28^**^ | .32^**^ | .39^**^ | .35^**^ | .39^**^ | .50^**^ | .56^**^ | .76^**^ | .56^**^ | .08^*^ | .11^**^ | .08^*^ | .11^**^ | .05 | .28^**^ | .19^**^ | .27^**^ | .33^**^ | .40^**^ | .05 | .49^**^ | .40^**^ | .54^**^ | .62^**^ | - |  |
| 28. Dep T6 | .18^**^ | .25^**^ | .34^**^ | .31^**^ | .28^**^ | .39^**^ | .31^**^ | .39^**^ | .45^**^ | .51^**^ | .80^**^ | .01 | .08 | .004 | .08 | .011 | .19^**^ | .23^**^ | .15^**^ | .26^**^ | .25^**^ | .37^**^ | .35^**^ | .32^**^ | .39^**^ | .47^**^ | .51^**^ | - |
| Mean | 2.25 | 2.29 | 2.37 | 2.37 | 2.56 | 2.30 | 2.15 | 2.28 | 2.23 | 2.30 | 2.20 | .23 | .24 | .28 | .27 | .21 | 7.13 | 7.09 | 7.06 | 6.90 | 6.86 | 6.72 | .14 | .14 | .15 | .15 | .15 | .15 |
| *SD* | .93 | .91 | .85 | .86 | .87 | .89 | 1.14 | 1.24 | 1.15 | 1.20 | 1.16 | .16 | .17 | .15 | .16 | .18 | 2.78 | 2.65 | 2.67 | 2.44 | 2.75 | 2.22 | .13 | .13 | .14 | .14 | .14 | .14 |

*Note.*  NSSI = non-suicidal self-injury; Friend stress = friendship stress; Lon = loneliness; Vict = peer victimization; Dep = depressive symptoms. For peer victimization log_10_ values are reported.

**p* <.05; ***p* <.01

**Appendix S4. Random Intercept Cross-Lagged Panel Model (RI-CLPM) between Friendship Stress and Non-Suicidal Self-Injury**

We examined time-invariance of the main parameters by comparing a model with all parameters freely estimated across time (Model 1) to a model in which, each time, groups of parameters were constrained to be similar (i.e., invariant) over time. Models were compared using chi-square difference tests (*Δχ*2). First, we examined whether the autoregressive paths of friendship stress (Model 2) and NSSI (Model 3) could be constrained to be equal over time. These equalities constraints worsened the mode fit (see Table S2). Second, we examined whether concurrent associations could be constrained to be equal over time (Model 4); also, these constraints worsened the model fit. Finally, we examined whether cross-lagged effects could be constrained to be equal over time, separately from friendship stress to NSSI (Model 5) and from NSSI to friendship stress (Model 6). Adding equality constrained over time to the cross-lagged effects did not worsened the model fit in both cases. Therefore, in the final model (Model 7) the autoregressive paths and the concurrent associations (i.e., within-time residual covariances and variances) were freely estimated over time, whereas all cross-lagged effects were fixed to be equal over time. This model had a good fit to the data (Table S2) and fitted the data equally well as the model with all freely estimated parameters (Model 1). Therefore, this model (Model 7) was retained as final model.

**Table S2**

*Model Fit Indices and Model Fit Comparisons of Random-Intercept Cross-Lagged Panel Models between Friendships Stress and Non-Suicidal Self-Injury*

|  | Model fit indices | | | | | |  | Difference test | | |
| --- | --- | --- | --- | --- | --- | --- | --- | --- | --- | --- |
| Models | χ^2^ | *df* | *p* | CFI | TLI | RMSEA |  | Δχ^2^ | Δ*df* | *p* |
| Model 1 | 41.735 | 37 | 0.273 | 0.997 | 0.995 | 0.012 |  |  |  |  |
| Model 2 | 49.826 | 41 | 0.162 | 0.995 | 0.992 | 0.016 |  | 10.410 | 4 | 0.034 |
| Model 3 | 53.196 | 41 | 0.096 | 0.993 | 0.989 | 0.019 |  | 12.108 | 4 | 0.016 |
| Model 4 | 64.048 | 49 | 0.073 | 0.992 | 0.989 | 0.019 |  | 25.035 | 12 | 0.015 |
| Model 5 | 44.173 | 41 | 0.339 | 0.998 | 0.997 | 0.009 |  | 2.765 | 4 | 0.598 |
| Model 6 | 45.344 | 41 | 0.296 | 0.998 | 0.996 | 0.011 |  | 4.265 | 4 | 0.371 |
| **Model 7** | **47.187** | **45** | **0.383** | **0.999** | **0.998** | **0.007** |  | **5.805** | **8** | **0.669** |

*Note*. Weighted Least Square Mean and Variance adjusted estimator (WLSMV) was used; therefore, the chi-square for difference testing (Δχ^2^) was used for comparing nested models. Model 7 (i.e., in bold) is the final model.

**Appendix S5. Random Intercept Cross-Lagged Panel Model (RI-CLPM) between Loneliness and Non-Suicidal Self-Injury**

The same procedure used for the models including friendship stress was followed. First, fixing the autoregressive paths of loneliness to be equal over time (Model 2) did not worsen the model fit (see Table S6). However, a model with constrains on the autoregressive paths of NSSI (Model 3) yielded a poorer model fit (see Table S3). In Model 4, fixing the concurrent associations (i.e., within-time residual covariances and variances) to be equal over time also worsened the model fit. Finally, the cross-lagged effects emerged to be equal over time, both from loneliness to NSSI (Model 5) as well as from NSSI to loneliness (Model 6). Therefore, in the final model (Model 7) the autoregressive paths for NSSI and the concurrent associations were freely estimated over time; However, the autoregressive paths for loneliness and all cross-lagged effects were fixed to be equal over time. This model had a good fit to the data (Table S3) and fitted the data equally well as the model with all freely estimated parameters (Model 1). Therefore, this model (Model 7) was retained as final model.

**Table S3**

*Model Fit Indices and Model Fit Comparisons of Random-Intercept Cross-Lagged Panel Models between Loneliness e Non-Suicidal Self-Injury*

|  | Model fit indices | | | | | |  | Difference test | | |
| --- | --- | --- | --- | --- | --- | --- | --- | --- | --- | --- |
| Models | χ^2^ | *df* | *p* | CFI | TLI | RMSEA |  | Δχ^2^ | Δ*df* | *p* |
| Model 1 | 31.730 | 21 | 0.062 | .991 | .982 | .025 |  |  |  |  |
| Model 2 | 33.861 | 24 | 0.087 | .992 | .985 | .022 |  | 1.441 | 3 | 0.696 |
| Model 3 | 42.704 | 24 | 0.011 | .985 | .972 | .031 |  | 10.630 | 3 | 0.014 |
| Model 4 | 49.688 | 30 | 0.013 | .984 | .976 | .028 |  | 19.552 | 9 | 0.021 |
| Model 5 | 37.418 | 24 | 0.039 | .989 | .980 | .026 |  | 6.812 | 3 | 0.079 |
| Model 6 | 31.920 | 24 | 0.129 | .994 | .988 | .020 |  | 1.177 | 3 | 0.759 |
| **Model 7** | **35.651** | **30** | **0.219** | **.995** | **.993** | **.015** |  | **7.735** | **9** | **0.561** |

*Note*. Weighted Least Square Mean and Variance adjusted estimator (WLSMV) was used; therefore, the chi-square for difference testing (Δχ^2^) was used for comparing nested models. Model 7 (i.e. in bold) is the final model.

**Appendix S6. Random Intercept Cross-Lagged Panel Model (RI-CLPM) between Peer Victimization and NSSI**

The same procedure of friendship stress and loneliness was used. Model 2 examined whether the autoregressive paths of peer victimization could be constrained to be equal over time; this worsened the model fit, so these paths were freely estimated over time. Subsequently a model with constrains on the autoregressive paths of NSSI (Model 3) was examined. This model did not worsen the model fit, so these constraints were retained (see Table S4). In Model 4, it was examined whether concurrent associations (i.e., within-time residual covariances and variances) could be constrained to be equal over time; these constraints worsened the model fit, so these paths were freely estimated over time. Subsequently, it was examined whether cross-lagged effects could be fixed to be equal over time separately from peer victimization to NSSI (Model 5) and from NSSI to peer victimization (Model 6). Adding equality constrained over time to the cross-lagged effects did not worsened the model fit in both cases. Therefore, in the final model (Model 7) the autoregressive paths for peer victimization and the concurrent associations were freely estimated over time, whereas the autoregressive paths for NSSI and all cross-lagged effects were fixed to be equal over time. This model had a good fit to the data (Table S4) and fitted the data equally well as the model with all freely estimated parameters (Model 1). Therefore, this model (Model 7) was retained as final model.

**Table S4**

*Model Fit Indices and Model Fit Comparisons of Random-Intercept Cross-Lagged Panel Models between Peer Victimization and Non-Suicidal Self-Injury*

|  | Model fit indices | | | | | |  | Difference test | | |
| --- | --- | --- | --- | --- | --- | --- | --- | --- | --- | --- |
| Models | χ^2^ | *df* | *p* | CFI | TLI | RMSEA |  | Δχ^2^ | Δ*df* | *p* |
| Model 1 | 32.141 | 21 | 0.057 | 0.991 | 0.982 | 0.025 |  |  |  |  |
| Model 2 | 66.099 | 24 | <.001 | 0.968 | 0.939 | 0.046 |  | 37.208 | 3 | <.001 |
| Model 3 | 37.137 | 24 | 0.042 | 0.990 | 0.981 | 0.026 |  | 5.832 | 3 | 0.120 |
| Model 4 | 58.653 | 30 | 0.001 | 0.978 | 0.967 | 0.034 |  | 29.831 | 9 | <.001 |
| Model 5 | 36.547 | 24 | 0.048 | 0.990 | 0.982 | 0.025 |  | 5.774 | 3 | 0.123 |
| Model 6 | 37.051 | 26 | 0.074 | 0.991 | 0.985 | 0.023 |  | 4.091 | 3 | 0.252 |
| **Model 7** | **45.209** | **30** | **0.037** | **0.988** | **0.982** | **0.025** |  | **14.555** | **9** | **0.104** |

*Note*. Weighted Least Square Mean and Variance adjusted estimator (WLSMV) was used; therefore, the chi-square for difference testing (Δχ^2^) was used for comparing nested models. Model 7 (i.e., in bold) is the final model.

**Table S5**

*Within-Person Effects on the Final Model (Model 7) between Friendship Stress and Non-Suicidal Self-Injury*

|  | Grade 7 | |  | Grade 8 | |  | Grade 9 | |  | Grade 10 | |  | Grade 11 | |  | Grade 12 | |
| --- | --- | --- | --- | --- | --- | --- | --- | --- | --- | --- | --- | --- | --- | --- | --- | --- | --- |
| Parameters | β (SE) | 95% C.I. |  | β (SE) | 95% C.I. |  | β (SE) | 95% C.I. |  | β (SE) | 95% C.I. |  | β (SE) | 95% C.I. |  | β (SE) | 95% C.I. |
| Within-person  correlations | .45 (.10)*** | .20-.70 |  | .32 (.08)*** | .10-.53 |  | .17 (.07)* | -.01-.34 |  | .16 (.08)* | -.04-.35 |  | .15 (.08) | -.07-.36 |  | .06 (.07) | -.13-.25 |
| Friendship stress  🡪 NSSI |  |  |  | .04(.06) | -.12-.19 |  | .03 (.05) | -.10-.16 |  | .03 (.04) | -.09-.14 |  | .03 (.05) | -.09-.15 |  | .02(.03) | -.06-.10 |
| NSSI 🡪  Friendship stress |  |  |  | .11 (.05)* | -.01-.23 |  | .10 (.04)* | -.01-.22 |  | .12 (.05)* | -.01-.25 |  | .14 (.06)* | -.01-.28 |  | .12 (.05)* | -.01-.25 |
| Autoregressive paths  friendship stress |  |  |  | .37 (.05)*** | .23-.50 |  | .33 (.05)*** | .20-.47 |  | .33 (.06)*** | .18-.47 |  | .24 (.06)*** | .08-.40 |  | .15 (.06)** | .01-.30 |
| Autoregressive  paths NSSI |  |  |  | .41 (.10)*** | .15-.67 |  | .35 (.10)*** | .10-.61 |  | .45 (.06)*** | .28-.61 |  | .58 (.06)*** | .43-.72 |  | .47 (.08)*** | .27-.67 |

*Note.* The between-person effect is not reported in the table; this was β (SE)= .40 (.07), 95% C.I.= .22-.58, *p* < .001. Estimates for the cross-lagged and autoregressive effects reported in columns “Grade 8” to “Grade 12” refer to the grade in which the outcomes were measured; for example, longitudinal effects from grade 7 to grade 8 are reported in column “Grade 8”.

**p* <.05; ***p* <.01; ****p* <.001

**Appendix S7. Reciprocal Associations between Friendship Stress and NSSI at the Within-Person Level**

Results from the final RI-CLPM indicated that, in addition to the significant cross-lagged effects from NSSI to friendship stress, the concurrent correlations emerged to be significant. This suggests that when adolescents reported higher level of friendships stress (as compared to their own mean level) at a specific time point, they also reported more NSSI at that time point (i.e., from Grade 7 to Grade 10, see Table S5). Moreover, the autoregressive paths of both friendships stress and NSSI were all positive and significant, indicating that adolescents tended to report higher levels of NSSI and friendship stress (as compared to their own mean levels) when they also reported higher levels of NSSI and friendship stress respectively, at the previous assessment (i.e., carry-over effect).

**Table S6**

*Within-Person Effects on the Final Model (Model 7) between Loneliness and Non-Suicidal Self-Injury*

|  | Grade 8 | |  | Grade 9 | |  | Grade 10 | |  | Grade 11 | |  | Grade 12 | |
| --- | --- | --- | --- | --- | --- | --- | --- | --- | --- | --- | --- | --- | --- | --- |
| Parameters | β (SE) | 95% C.I. |  | β (SE) | 95% C.I. |  | β (SE) | 95% C.I. |  | β (SE) | 95% C.I. |  | β (SE) | 95% C.I. |
| Within-person  correlations | .39 (.10)*** | .13-.66 |  | .34 (.07) *** | .17-.51 |  | .41 (06) *** | .26-.55 |  | .14 (.08) | -.06-.35 |  | .30 (.08)*** | .11-.50 |
| Loneliness  🡪 NSSI |  |  |  | .01 (.05) | -.11-.14 |  | .01 (.05) | -.12-.15 |  | .02 (.06) | -.13-.16 |  | .01 (.04) | -.09-.11 |
| NSSI 🡪  Loneliness |  |  |  | .13 (.05)** | .01-.25 |  | .15 (.05)** | .01-.28 |  | .16 (.06)** | .01-31 |  | .15 (.05)** | .01-.29 |
| Autoregressive paths  Loneliness |  |  |  | .34 (.04)*** | .23-.46 |  | .39 (.06)*** | .23-.54 |  | .40 (.07)*** | .24-.57 |  | .39 (.07)*** | .22-57 |
| Autoregressive  paths NSSI |  |  |  | .48 (.09)*** | .24-.72 |  | .56 (.07)*** | .38-.75 |  | .65 (.06)*** | .49-.81 |  | .53 (.07)*** | .34-.71 |

*Note.* The between-person effect is not report in the table; this was β (SE)=.68 (.08); 95% C.I.= .48-.88. Estimates for the cross-lagged and autoregressive effects reported in columns “Grade 9” to “Grade 12” refer to the grade in which the outcomes were measured; for example, longitudinal effects from grade 8 to grade 9 are reported in column “Grade 9”.

**p* <.05; ***p* <.01; ****p* <.001

**Figure S1**

*
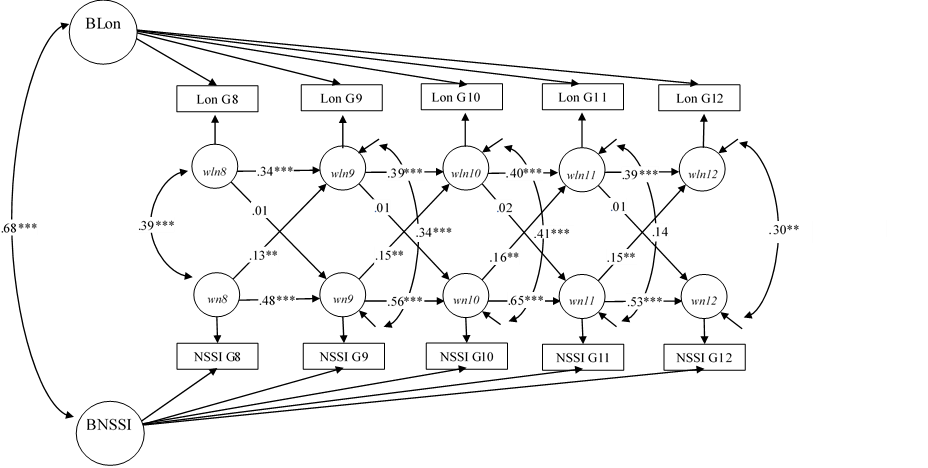
Final Random-Intercept Cross-lagged Panel Model between Loneliness and NSSI.*

*Note.*  BLon= Between-person random intercept for loneliness; BNSSI= Between-person random intercept for NSSI; LonG8-LonG12= observed loneliness scores from Grade 8 to 12; NSSIG8- NSSIG12= observed NSSI scores from Grade 8 to 12; *wln8-wln12* = within-person latent factors for loneliness from Grade 8 to 12; *wn8-wn12* = within-person latent factors for NSSI from Grade 8 to 12. Standardized estimates are reported. Model fit: χ² (30) = 35.651 (*p* = .219), RMSEA = .015 (90% *CI* = [.000, .032]), CFI = 0.995, TLI = 0.993, and WRMR= 0.454.

**p* <.05; ***p* <.01; ****p* <.001

**Appendix S8. Reciprocal Associations between Loneliness and NSSI at the Within-Person Level**

Results from the final RI-CLPM indicated that, in addition to the significant cross-lagged effects from NSSI to loneliness, the concurrent correlations were also significant, indicating that when adolescents reported higher level of loneliness (as compared to their own mean level) at a specific time point, they also reported more NSSI at that same time point. This was the case for all time points, with the exception of Grade 11. The autoregressive paths of both loneliness and NSSI were all positive and significant, indicating that adolescents tended to report higher levels of loneliness and NSSI (as compared to their own mean levels) when they also reported higher levels of loneliness and NSSI respectively, at the previous assessment.

**Table S7**

*Within-Person Effects on the Final Model (Model 7) between Peer Victimization and NSSI*

|  | Grade 8 | |  | Grade 9 | |  | Grade 10 | |  | Grade 11 | |  | Grade 12 | |
| --- | --- | --- | --- | --- | --- | --- | --- | --- | --- | --- | --- | --- | --- | --- |
| Parameters | β (SE) | 95% C.I. |  | β (SE) | 95% C.I. |  | β (SE) | 95% C.I. |  | β (SE) | 95% C.I. |  | β (SE) | 95% C.I. |
| Within-person  correlations | .24 (.12)* | -.08 -.56 |  | .10 (.07) | -.09-.29 |  | .-.13 (.08) | -.34-.08 |  | -.01 (.08) | -.22-.20 |  | -.18 (.04)* | -.39-.04 |
| Peer victimization  🡪 NSSI |  |  |  | .05 (.03) | -.03-.14 |  | .07 (.04) | -.04-.19 |  | .07 (.04) | -.04-.19 |  | .06 (.03) | -.03-.14 |
| NSSI 🡪  Peer victimization |  |  |  | .16 (.05)** | .02-29 |  | .17 (.06)** | .03-31 |  | .18 (.06)** | .03-.33 |  | .17 (.06)** | .03-.31 |
| Autoregressive paths  Peer victimization |  |  |  | .31 (.06)*** | .16-.46 |  | .44 (.04)*** | .33-.56 |  | .32 (.05)*** | .18-.46 |  | .02 (.06) | -.12-.17 |
| Autoregressive  paths NSSI |  |  |  | .71 (.09)*** | .48-.94 |  | .73 (.08)*** | .52-.93 |  | .77 (.06)*** | .60-.93 |  | .59 (.08)*** | .39-.79 |

*Note.* The between-person effect is not report in the table; this was β (SE)= -.22 (1.18); 95% C.I.=-3.27-2.82. Estimates for the cross-lagged and autoregressive effects reported in columns “Grade 9” to “Grade 12” refer to the grade in which the outcomes were measured; for example, longitudinal effects from grade 8 to grade 9 are reported in column “Grade 9”.

**p* <.05; ***p* <.01; ****p* <.001

**Figure S2**


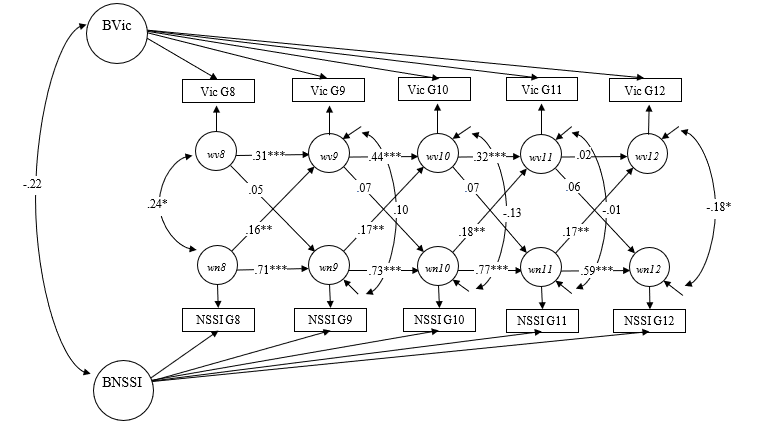
*Final Random-Intercept Cross-lagged Panel Model between Peer Victimization and NSSI.*

*Note.*  BVic= Between-person random intercept for peer victimization; BNSSI= Between-person random intercept for NSSI; VicG8- VicG12= observed peer victimization scores from Grade 8 to 12; NSSIG8- NSSIG12= observed NSSI scores from Grade 8 to 12; *wv8-wv12* = within-person latent factors for peer victimization from Grade 8 to 12; *wn8-wn12* = within-person latent factors for NSSI from Grade 8 to 12. Standardized estimates are reported. Model fit: χ² (30) = 45.209 (*p* = .037), RMSEA = .025 (90% *CI* = [.006, .039]), CFI = 0.988, TLI = 0.982, and WRMR= 0.672.

**p* <.05; ***p* <.01; ****p* <.001

**Appendix S9.** **Reciprocal Associations between Peer Victimization and NSSI at the Within-Person Level**

Results from the final RI-CLPM indicated that, in addition to the significant cross-lagged effects from NSSI to peer victimization, significant concurrent correlations were found at Grade 8 and 12. At Grade 8, this association was positive, indicating that higher than usual peer victimization levels were associated with higher than usual NSSI levels; yet, the association was negative at Grade 12, suggesting an opposite effect. Besides, the autoregressive paths of peer victimization were also positive and significant, except from Grade 11 to Grade 12, while for NSSI they were significant across all time points.

**Appendix S10. Cross-lagged Panel Models (CLPM) between Peer Problems and Non-Suicidal Self-Injury**

We compared time-variant CLPMs, with all parameters freely estimated over time (CLPM, Model 1), to the RI-CLPMs with all parameters freely estimated over time (RI-CLPM, Model 1). Moreover, we compared the final, retained RI-CLPMs (Models 7), with CLPMs in which the same parameters were constrained to be equal over time (CLPM, Models 7). As shown in Table S9, all RI-CLPMs fitted the data better than CLPMs. All models reported in Figures S1-S3 are CLPMs with estimates fixed to be equal over time (Models 7).

**Table S8**

*Model Fit Indices and Model Fit Comparisons of Cross-lagged Panel Models between Peer Problems and Non-Suicidal Self-Injury*

|  | Model fit indices | | | | | |  | Difference test | | |
| --- | --- | --- | --- | --- | --- | --- | --- | --- | --- | --- |
| Models | χ^2^ | df | p | CFI | TLI | RMSEA |  | *Δχ^2^* | *Δdf* | *p* |
| *Friendship stress* |  |  |  |  |  |  |  |  |  |  |
| RI-CLPM, Model 1 | 41.735 | 37 | 0.273 | 0.997 | 0.995 | 0.012 |  |  |  |  |
| CLPM, Model 1 | 209.519 | 40 | <.001 | 0.906 | 0.845 | 0.070 |  | 147.156 | 3 | <.001 |
| RI-CLPM, Model 7 | 47.187 | 45 | 0.383 | 0.999 | 0.998 | 0.007 |  |  |  |  |
| CLPM, Model 7 | 169.262 | 48 | <.001 | 0.933 | 0.908 | 0.054 |  | 122.011 | 3 | <.001 |
| *Loneliness* |  |  |  |  |  |  |  |  |  |  |
| RI-CLPM, Model 1 | 31.730 | 21 | 0.062 | 0.991 | 0.982 | 0 .025 |  |  |  |  |
| CLPM, Model 1 | 84.187 | 24 | <.001 | 0.952 | 0.910 | 0.055 |  | 38.359 | 3 | <.001 |
| RI-CLPM, Model 7 | 35.651 | 30 | 0.219 | 0.992 | 0.986 | 0.022 |  |  |  |  |
| CLPM, Model 7 | 67.598 | 33 | <.001 | 0.972 | 0.962 | 0.036 |  | 36.919 | 3 | <.001 |
| *Peer Victimization* |  |  |  |  |  |  |  |  |  |  |
| RI-CLPM, Model 1 | 32.141 | 21 | 0.057 | 0.991 | 0.982 | 0.025 |  |  |  |  |
| CLPM, Model 1 | 157.112 | 24 | <.001 | 0.897 | 0.808 | 0.082 |  | 74.439 | 3 | <.001 |
| RI-CLPM, Model 7 | 45.209 | 30 | 0.037 | 0.988 | 0.982 | 0.025 |  |  |  |  |
| CLPM, Model 7 | 119.594 | 33 | <.001 | 0.933 | 0.909 | 0.056 |  | 79.450 | 3 | <.001 |

*Note*. Weighted Least Square Mean and Variance adjusted estimator (WLSMV) was used; therefore, the chi-square for difference testing (Δχ^2^) was used.

**Figure S3**

*Diagram of Final Cross-Lagged Panel Models between Friendship Stress and Non-Suicidal Self-Injury.*

.70***

.68***04

.76***34

.77***04

.73***84

Fs G12

Fs G7

Fs G8

Fs G11

Fs G10

Fs G9

.34***

.14

.27**

.23**

.004

.0044

.003

.002

.003

.05

.42***4

.04*

.04*

.05*

.04*

.05*

.68******

.76***

NSSI G12

NSSI G11

NSSI G7

NSSI G8

NSSI G9

NSSI G10

.82***

.79***

.74***

*Note.* FS= friendship stress; NSSI= non-suicidal self-injury; G= School grade. Standardized estimates are reported.

**p* <.05; ***p* <.01; ****p* <.001

**Figure S4**

*Diagram of Final Cross-Lagged Panel Models between Loneliness and Non-Suicidal Self-Injury.*

Lon G10

Lon G12

Lon G9

Lon G8

Lon G11

NSSI G10

NSSI G12

NSSI G9

NSSI G8

NSSI G11

.54***4

.72***84

.75***04

.76***34

.72***

.68***

.73***

.63******

.77***

.05

.05

.05

.04

.08***7

.08***

.09***

.08***

.32***

.34***

.44***4

.11

*Note.* Lon= loneliness; NSSI= non-suicidal self-injury; G= School grade. Standardized estimates are reported.

**p* <.05; ***p* <.01; ****p* <.001

**Figure S5**

*Diagram of Final Cross-Lagged Panel Models between Peer Victimization and Non-Suicidal Self-Injury.*

Vic G10

Vic G12

Vic G9

Vic G8

Vic G11

NSSI G10

NSSI G12

NSSI G9

NSSI G8

NSSI G11

.20***4

.70***84

.79***04

.72***34

.51***

.72***

.76***

.62******

.79***

.03

.03

.03

.02

.04

.04

.04

.04

-.18*

.08

-.10

.02

*Note.* Vic= peer victimization; NSSI= non-suicidal self-injury; G= School grade. Standardized estimates are reported.

**p* <.05; ***p* <.01; ****p* <.001

**Appendix S11. Gender Moderation Analysis**

Gender differences were examined using a multi-group approach. Specifically, a model with all parameters freely estimated across gender (Model 1) was compared to a constrained model in which cross-lagged effects were fixed to be equal for boys and girls (Model 2), using the chi-square difference test (*Δχ*2). Results from model comparisons are reported in Table S8.

**Table S9**

*Model Fit Indices and Model Fit Comparisons for Gender Multi-Group Random-Intercept Cross-lagged Panel Models.*

|  | Model fit indices | | | | | |  | Difference test | | |
| --- | --- | --- | --- | --- | --- | --- | --- | --- | --- | --- |
| Models | χ^2^ | df | p | CFI | TLI | RMSEA |  | Δχ^2^ | Δ*d* | *p* |
| *Friendship stress* | | | | | | | | | | |
| Model 1 | 95.648 | 78 | 0.085 | 0.989 | .0982 | 0.023 |  |  |  |  |
| Model 2 | 95.274 | 88 | 0.278 | 0.996 | 0.993 | 0.014 |  | 3.827 | 10 | 0 .955 |
| *Loneliness* | | | |  |  |  |  |  |  |  |
| Model 1 | 48.353 | 42 | 0.232 | 0.995 | 0.989 | 0.019 |  |  |  |  |
| Model 2 | 53.386 | 50 | 0.346 | 0.997 | 0.995 | 0.013 |  | 5.75 | 8 | 0.675 |
| *Peer victimization* | | | | | | | | | | |
| Model 1 | 50.458 | 46 | 0.302 | 0.996 | 0.993 | 0.015 |  |  |  |  |
| Model 2 | 54.531 | 54 | 0.454 | 1.000 | 0.999 | 0.005 |  | 6.41 | 8 | 0.602 |

*Note*. Weighted Least Square Mean and Variance adjusted estimator (WLSMV) was used; therefore, the chi-square for difference testing (Δχ^2^) was used for comparing nested models.

**Appendix S12. Random Intercept Cross-Lagged Panel Model (RI-CLPM) with Friendship Stress, Depression and NSSI**

Similarly, to the main RI-CLPMs, also in all models including depressive symptoms we examined time-invariance of parameter estimates. First, we examined whether the autoregressive paths of friendship stress (Model 2), depression (Model 3) and NSSI (Model 4) could be constrained to be equal over time, by comparing these models to a model with all parameters freely estimated over time (Model 1). The constraints on the autoregressive paths of friendship stress and depression did not worsened the model fit; however, constraining the autoregressive paths of NSSI to be equal over time worsened the model fit (see Table S10). Then, we examined whether the concurrent associations (i.e., within-time residual covariances and variances) (Model 5) could be constrained to be equal over time (note that time-invariance of all within-time residual covariances and variances was tested in one-step). These equalities constraints worsened the model fit (see Table S10). Next, we examined whether cross-lagged effects could be constrained to be equal over time, separately from NSSI to friendship stress (Model 6), from friendship stress to NSSI (Model 7), from depression to friendship stress (Model 8), from friendship stress to depression (Model 9), from depression to NSSI (Model 10) and from NSSI to depression (Model 11). Adding equality constrained over time to the cross-lagged effects did not worsened the model fit, with the exception of the cross-lagged from friendship stress to depression. Therefore, in the final model (Model 12) the autoregressive paths for NSSI, the concurrent associations (i.e., within-time residual covariances and variances) and the cross-lagged effects from friendship stress to depression were freely estimated over time, all other parameters were fixed to be equal over time. This model had a good fit to the data (Table S10) and fitted the data equally well as the model with all freely estimated parameters (Model 1). Thus, this model (Model 12) was retained as final model and is displayed in Figures S6.

|  | Model fit indices | | | | | |  | Difference test | | |
| --- | --- | --- | --- | --- | --- | --- | --- | --- | --- | --- |
| Models | χ^2^ | *df* | *p* | CFI | TLI | RMSEA |  | Δχ^2^ | Δ*df* | *p* |
| Model 1 | 89.707 | 84 | .315 | .998 | .996 | .009 |  |  |  |  |
| Model 2 | 96.252 | 88 | .257 | .997 | .995 | .010 |  | 8.286 | 4 | .082 |
| Model 3 | 94.442 | 88 | .300 | .998 | .996 | .009 |  | 6.622 | 4 | .157 |
| Model 4 | 101.137 | 88 | .160 | .995 | .991 | .013 |  | 14.018 | 4 | .007 |
| Model 5 | 128.519 | 108 | .087 | .992 | .989 | .015 |  | 40.122 | 24 | .021 |
| Model 6 | 93.592 | 88 | .322 | .998 | .996 | .009 |  | 4.452 | 4 | .348 |
| Model 7 | 92.408 | 88 | .353 | .998 | .997 | .008 |  | 2.756 | 4 | .559 |
| Model 8 | 92.462 | 88 | .352 | .998 | .997 | .008 |  | 1.932 | 4 | .748 |
| Model 9 | 99.202 | 88 | .195 | .996 | .993 | .012 |  | 12.795 | 4 | .012 |
| Model 10 | 94.208 | 88 | .306 | .998 | .996 | .009 |  | 5.300 | 4 | .258 |
| Model 11 | 92.954 | 88 | .338 | .998 | .997 | .008 |  | 4.502 | 4 | .342 |
| **Model 12** | **118.096** | **112** | **.328** | **.998** | **.997** | **.008** |  | **29.451** | **28** | **.390** |

**Table S10**

*Model Fit Indices and Model Fit Comparisons of Random-Intercept Cross-Lagged Panel Models with Friendships Stress, Depression and Non-Suicidal Self-Injury*

**Figure S6**

*Final Between- and Within-Person Effect of the Random-Intercept Cross-lagged Panel Model with Friendship Stress, Depression and NSSI*

.48***

.33***

.15*

.15

.12

.43***

.39***

.44***

.60***

.45***

.33***

.31***

.32***

.28**

.05

.04

.04

.04

.02

.09 †

.08 †

.10 †

.11 †

.11 †

.30***

.05

.46***

.43***

.26***

.28***

.15**

.30***

.28***

.30***

.34***

.31***

-.03

.20***

.19**

.01

-.02

.08*

.09*

.10*

.11*

.10*

.22***

.33***

.31***

.32***

.28**

.30***

.44***

.41***

.40***

.42***

.28***

.02

.02

.02

.02

.02

.14**

.10*

.12**

.13**

.12**

.46***

.43***

.39***

.44***

.60***

.45***

.30***

.28***

.30***

.34***

.31***

.64***

.66***

.38***

*Note.* BFs= Between-person random intercept for friendship stress; BNSSI= Between-person random intercept for NSSI; BDep= Between-person random intercept for depression. wfs7-wfs12 = within-person latent factors for friendship stress from Grade 7 to 12. wnssi7-wnnsi12 = within-person latent factors for NSSI from Grade 7 to 12. wd7-wd12 = within-person latent factors for depression from Grade 7 to 12. Standardized estimates are reported. **p* <.05; ***p* <.01; ****p* <.001. †<.07

**Appendix S13. Random Intercept Cross-Lagged Panel Model (RI-CLPM) with Loneliness, Depression and NSSI**

The same procedure used for the models with friendship stress was followed. First, fixing the autoregressive paths of loneliness (Model 2) and depression (Model 3) to be equal over time did not worsen the model fit (see Table S11). However, a model with constrains on the autoregressive paths of NSSI (Model 4) did not converge; thus, it was decided to leave these paths free over time. Subsequently, as for the model with friendship stress, we examined whether the concurrent associations (i.e., within-time residual covariances and variances) (Model 5) could be all constrained to be equal over time. These equalities constraints worsened the model fit (see Table S11). Finally, all cross-lagged effects emerged to be time-invariant (see Models 6-11). Therefore, in the final model (Model 12) the autoregressive paths for NSSI and the concurrent associations were freely estimated over time, whereas the autoregressive paths for loneliness and depression as well as all cross-lagged effects were fixed to be equal over time. This model had a good fit to the data (Table S11) and fitted the data equally well as the model with all freely estimated parameters (Model 1). Thus, this model (Model 12) was retained as final model and is displayed in Figures S7.

**Table S11**

*Model Fit Indices and Model Fit Comparisons of Random-Intercept Cross-Lagged Panel Models with Loneliness, Depression and Non-Suicidal Self-Injury*

|  | Model fit indices | | | | | |  | Difference test | | |
| --- | --- | --- | --- | --- | --- | --- | --- | --- | --- | --- |
| Models | χ^2^ | *df* | *p* | CFI | TLI | RMSEA |  | Δχ^2^ | Δ*df* | *p* |
| Model 1 | 66.070 | 48 | .043 | .991 | .980 | .021 |  |  |  |  |
| Model 2 | 68.123 | 51 | .055 | .991 | .982 | .020 |  | 1.469 | 3 | .689 |
| Model 3 | 69.233 | 51 | .046 | .991 | .980 | .021 |  | 3.667 | 3 | .299 |
| Model 4 | - | - | - | - | - | - | - | - | - | - |
| Model 5 | 102.912 | 66 | .003 | .982 | .971 | .026 |  | 38.358 | 18 | .004 |
| Model 6 | 66.746 | 51 | .069 | .992 | .984 | .019 |  | 1.066 | 3 | .785 |
| Model 7 | 68.173 | 51 | .054 | .991 | .980 | .021 |  | 2.212 | 3 | .529 |
| Model 8 | 69.332 | 51 | .045 | .991 | .981 | .021 |  | 5.558 | 3 | .135 |
| Model 9 | 67.571 | 51 | .060 | .992 | .983 | .020 |  | 1.622 | 3 | .654 |
| Model 10 | 68.828 | 51 | .049 | .991 | .982 | .021 |  | 4.658 | 3 | .199 |
| Model 11 | 70.135 | 51 | .039 | .990 | .980 | .021 |  | 4.554 | 3 | .208 |
| **Model 12** | **77.053** | **72** | **.320** | **.997** | **.996** | **.009** |  | **20.143** | **24** | **.689** |

*Note*. Model 4 did not converge

**Figure S7**

*Final Between- and Within-Person Effect of the Random-Intercept Cross-lagged Panel Model with Loneliness, Depression and NSSI*

.43***

.33***

.43***

.15*

.32***

.51***

.57***

.69***

.52***

.28***

.30***

.29***

-.004

-.004

-.004

-.003

.08

.09

.09

.09

.26***

.61***

.65***

.62***

.53***

.65***

.26***

.29***

.31***

.29***

.12*

.13*

.15*

.13**

.19***

.20***

.22***

.20***

.28***

.30***

.29***

.26***

.43***

.41***

.44***

26**

.46***

.07

.08

.09

.08

.51***

.57***

.69***

.52***

.90***

.76***

.72***

.29***

.31***

.29***

.26***

*Note.* BLn= Between-person random intercept for loneliness; BNSSI= Between-person random intercept for NSSI; BDep= Between-person random intercept for depression. wln8-wfs12 = within-person latent factors for friendship stress from Grade 8 to 12. wnssi8-wnnsi12 = within-person latent factors for NSSI from Grade 8 to 12. wd8-wd12 = within-person latent factors for depression from Grade 8 to 12. Standardized estimates are reported. **p* <.05; ***p* <.01; ****p* <.001

-.004

-.006

-.006

-.005

**Appendix S14. Random Intercept Cross-Lagged Panel Model (RI-CLPM) with Peer Victimization, Depression and NSSI**

The same procedure used for the models with friendship stress and loneliness was followed. First, fixing the autoregressive paths of depression (Model 3) to be equal over time did not worsen the model fit (see Table S12). However, a model with constrains on the autoregressive paths of peer victimization (Model 2) and NSSI (Model 4) worsened the model fit. Next, constraining the concurrent associations (i.e., within-time residual covariances and variances) to be equal over time (Model 5) worsened the model fit (see Table S12). Finally, all cross-lagged effects, except the ones from NSSI to peer victimization (Model 6), emerged to be time-invariant (Models 7-11). Therefore, in the final model (Model 12) the autoregressive paths of peer victimization and NSSI, the concurrent associations and the cross-lagged effect from NSSI to peer victimization were freely estimated over time, whereas the autoregressive paths of depression and all other cross-lagged effects were fixed to be equal over time. This model had a good fit to the data (Table S12) and fitted the data equally well as the model with all freely estimated parameters (Model 1). Thus, this model (Model 12) was retained as final model and is displayed in Figures S8.

**Table S12**

*Model Fit Indices and Model Fit Comparisons of Random-Intercept Cross-Lagged Panel Models with Peer Victimization, Depression and Non-Suicidal Self-Injury*

|  | Model fit indices | | | | | |  | Difference test | | |
| --- | --- | --- | --- | --- | --- | --- | --- | --- | --- | --- |
| Models | χ^2^ | *df* | *p* | CFI | TLI | RMSEA |  | Δχ^2^ | Δ*df* | *p* |
| Model 1 | 63.879 | 48 | .062 | .992 | .982 | .020 |  |  |  |  |
| Model 2 | 98.201 | 51 | <.001 | .976 | .950 | .033 |  | 38.703 | 3 | <.001 |
| Model 3 | 67.655 | 51 | .059 | .991 | .982 | .020 |  | 4.685 | 3 | .196 |
| Model 4 | 72.077 | 51 | .028 | .989 | .978 | .022 |  | 9.746 | 3 | .030 |
| Model 5 | 96.372 | 66 | .009 | .984 | .975 | .023 |  | 34.218 | 18 | .012 |
| Model 6 | 72.816 | 51 | .024 | .989 | .977 | .023 |  | 7.887 | 3 | .048 |
| Model 7 | 67.447 | 51 | .061 | .992 | .983 | .020 |  | 4.824 | 3 | .185 |
| Model 8 | 67.668 | 51 | .059 | .991 | .982 | .020 |  | 4.204 | 3 | .240 |
| Model 9 | 64.120 | 51 | .103 | .993 | .986 | .018 |  | 1.830 | 3 | .608 |
| Model 10 | 68.669 | 51 | .050 | .991 | .981 | .020 |  | 6.631 | 3 | .085 |
| Model 11 | 67.083 | 51 | .065 | .992 | .983 | .019 |  | 3.560 | 3 | .313 |
| **Model 12** | **79.411** | **66** | **.124** | **.993** | **.989** | **.016** |  | **20.090** | **18** | **.328** |

**Figure S8**

*Final Between- and Within-Person Effect of the Random-Intercept Cross-lagged Panel Model with Peer Victimization, Depression and NSSI*

.41***

.45***

.56***

.60***

.73***

.55***

-.02

-.02

-.02

-.02

.12*

.13*

.14*

.13*

.07

-.04

-.14

.56***

.60***

.73***

.55***

.46***

.33***

.04

.05

.06

.06

.05

.19**

.07

.15 †

.00

.26***

.06

.09

-.02

.30***

.33***

.36***

.33***

.03

.04

.04

.04

.01

.01

.01

.01

.25**

.43***

.33***

.36***

.33***

.30***

.06

.07

-.08

*Note.* BVic= Between-person random intercept for peer victimization; BNSSI= Between-person random intercept for NSSI; BDep= Between-person random intercept for depression. wv8-wv12 = within-person latent factors for friendship stress from Grade 8 to 12. wnssi8-wnnsi12 = within-person latent factors for nssi from Grade 8 to 12. Wd8-wd12 = within-person latent factors for depression from Grade 8 to 12. Standardized estimates are reported. **p* <.05; ***p* <.01; ****p* <.001. †<.08

.07

.44***

.46***

.33***

.26***

.04

.13

.76***

.18

**References not in manuscript**

Asparouhov, T., Muthén, B., & Muthén, B. O. (2006). Robst chi square difference testing with mean and variance adjusted test statistics. *Matrix*, *1*(5), 1–6.

Bollen, K. A. (1989). A new incremental fit index for general structural equation models. *Sociological Methods & Research*, *17*(3), 303–316. https://doi.org/10.1177/0049124189017003004

Bollen, K. A., & Curran, P. J. (2006). *Latent curve models: A structural equation perspective* (Vol. 467). John Wiley & Sons.

Cillessen, A. H. N., & Marks, P. E. L. (2011). Conceptualizing and measuring popularity. In A. H. N. Cillessen, D. Schwartz, & L. Mayeux (Eds.), Popularity in the peer system (pp. 25–56). New York: Guilford Press.

Cillessen, A. H., & Marks, P. E. (2017). Methodological choices in peer nomination research. *New Directions for Child and Adolescent Development, 2017*(157), 21- 44. <https://doi.org/10.1002/cad.20206>

De Los Reyes, A., & Prinstein, M. J. (2004). Applying depression-distortion hypotheses to the assessment of peer victimization in adolescents. *Journal of Clinical Child and Adolescent Psychology, 33*(2), 325-335. <https://doi.org/10.1207/s15374424jccp3302_14.>

Hu, L., & Bentler, P. M. (1998). Fit indices in covariance structure modeling: Sensitivity to underparameterized model misspecification. *Psychological Methods*, *3*(4), 424-453. [https://doi.org/10.1037/1082-989X.3.4.424](https://psycnet.apa.org/doi/10.1037/1082-989X.3.4.424)

Little, R. J. (1988). A test of missing completely at random for multivariate data with missing values. *Journal of the American statistical Association*, *83*(404), 1198-1202.

Lasgaard, M. (2007). Reliability and validity of the Danish version of the UCLA Loneliness Scale. *Personality and Individual Differences, 42*(7), 1359-1366. <https://doi.org/10.1016/j.paid.2006.10.013>

Marks, P. E., Babcock, B., Cillessen, A. H., & Crick, N. R. (2013). The effects of participation rate on the internal reliability of peer nomination measures. Social Development, 22(3), 609-622. <https://doi.org/10.1111/j.1467-9507.2012.00661.x>

Mahon, N. E., Yarcheski, A., Yarcheski, T. J., Cannella, B. L., & Hanks, M. M. (2006). A meta-analytic study of predictors for loneliness during adolescence. *Nursing Research, 55*(5), 308-315. <https://doi.org/10.1097/00006199-200609000-00003>

Mulder, J. D., & Hamaker, E. L. (2021). Three extensions of the random intercept cross-lagged panel model. *Structural Equation Modeling: A Multidisciplinary Journal*, 1-11. <https://doi.org/10.1080/10705511.2020.1784738>

Schultzberg, M., Muthén, B., Asparouhov, T., & Hamaker, E. (2017). Dynamic structural equation modeling of intensive longitudinal data using multilevel time series analysis in Mplus version 8 part 7. *Workshop at Johns Hopkins University*.

Yu, C. Y. (2002). Evaluation of model fit indices for latent variable models with categorical and continuous outcomes. *MPlus software Web site*.
